# Transcriptional Programs Controlling Perinatal Lung Maturation

**DOI:** 10.1371/journal.pone.0037046

**Published:** 2012-08-20

**Authors:** Yan Xu, Yanhua Wang, Valérie Besnard, Machiko Ikegami, Susan E. Wert, Caleb Heffner, Stephen A. Murray, Leah Rae Donahue, Jeffrey A. Whitsett

**Affiliations:** 1 The Perinatal Institute and Section of Neonatology, Perinatal and Pulmonary Biology, University of Cincinnati, Cincinnati Children's Hospital Medical Center, Cincinnati, Ohio, United States of America; 2 The Jackson Laboratory, Bar Harbor, Maine, United States of America; University of Tübingen, Germany

## Abstract

The timing of lung maturation is controlled precisely by complex genetic and cellular programs. Lung immaturity following preterm birth frequently results in Respiratory Distress Syndrome (RDS) and Broncho-Pulmonary Dysplasia (BPD), which are leading causes of mortality and morbidity in preterm infants. Mechanisms synchronizing gestational length and lung maturation remain to be elucidated. In this study, we designed a genome-wide mRNA expression time-course study from E15.5 to Postnatal Day 0 (PN0) using lung RNAs from C57BL/6J (B6) and A/J mice that differ in gestational length by ∼30 hr (B6<A/J). Comprehensive bioinformatics and functional genomics analyses were used to identify key regulators, bioprocesses and transcriptional networks controlling lung maturation. We identified both temporal and strain dependent gene expression patterns during lung maturation. For time dependent changes, cell adhesion, vasculature development, and lipid metabolism/transport were major bioprocesses induced during the saccular stage of lung development at E16.5–E17.5. CEBPA, PPARG, VEGFA, CAV1 and CDH1 were found to be key signaling and transcriptional regulators of these processes. Innate defense/immune responses were induced at later gestational ages (E18.5–20.5), STAT1, AP1, and EGFR being important regulators of these responses. Expression of RNAs associated with the cell cycle and chromatin assembly was repressed during prenatal lung maturation and was regulated by FOXM1, PLK1, chromobox, and high mobility group families of transcription factors. Strain dependent lung mRNA expression differences peaked at E18.5. At this time, mRNAs regulating surfactant and innate immunity were more abundantly expressed in lungs of B6 (short gestation) than in A/J (long gestation) mice, while expression of genes involved in chromatin assembly and histone modification were expressed at lower levels in B6 than in A/J mice. The present study systemically mapped key regulators, bioprocesses, and transcriptional networks controlling lung maturation, providing the basis for new therapeutic strategies to enhance lung function in preterm infants.

## Introduction

Lung immaturity is a major cause of morbidity and mortality in premature infants and is commonly associated with RDS and BPD. Approximately 10% of all births (∼500,000 infants) are affected annually in the United States [1]. The risk of RDS is negatively correlated with the gestational age of the infant [2], [3]. Despite the impact of prematurity on related lung diseases following pre-term birth, mechanisms linking gestational length and lung maturation remain to be elucidated.

Lung development is a highly regulated and coordinated process typified by stage specific changes in structure and function that occur as the structural processes including branching morphogenesis, angiogenesis, sacculation, alveologenesis and cytodifferentiation [4], [5]. In mice, formation of the gas exchange region of the lung begins at approximately E15, and increases dramatically prior to birth. During this stage, the fetal lung undergoes remarkable structural, biochemical, and functional changes including sacculation and septation to form alveoli. Changes in lung structure are associated with increased cell differentiation and increased production of surfactant necessary for air breathing at birth [6]. The timing of lung maturation is likely controlled by complex cellular programs and is influenced by multiple factors (genetic, hormonal, and environmental). Using transgenic mice with conditional gene deletions/mutations in combination with functional genomics approaches, we and others have identified a number of transcription factors (TFs) that are critical for perinatal lung maturation, including NKX2.1 (NK2 homeobox 1), FOXA2 (forkhead box A2), GATA6 (GATA binding protein 6), NF-1 (neurofibromin 1), CEBPA (CCAAT/enhancer binding protein (C/EBP), alpha) and NFATC3 (nuclear factor of activated T-cells, cytoplasmic, calcineurin-dependent 3) [7], [8], [9], [10], [11], [12], [13], [14], [15]. These studies generally have focused to a single TF at a single developmental time. Less information is available regarding dynamic and combinatorial regulation of target genes by multiple TFs that are likely to mediate the complex process of perinatal lung maturation. We recently developed a systems approach integrating previous studies and identified the interactive nature of the factors that form a genetic network regulating surfactant homeostasis [16]. Murray et al. compared gestational lengths of 15 inbred mouse strains and found significant variability among inbred strains, while the intra-strain variation was low, supporting the concept that gestational length is genetically controlled [17], [18]. For example, A/J mice were born on embryonic (E) day 20.5 (492.3±0.9 hours) and B6 mice were born on E19.5 (462.4 hours±1.0 hours) respectively, a difference of 30 hours (P<0.0001) [17]. Besnard et al. further demonstrated that lung maturation proceeded more rapidly in short gestation B6 mice, and that B6 pups had better perinatal survival than A/J pups when born prematurely [19]. Maturation of lung structure, conducting and peripheral airway epithelial cell differentiation, and the induction of biochemical and genetic markers associated with pulmonary maturity occurred earlier in B6 mice. After ovarian transplantation, A/J pups were born early in B6 dams, demonstrating that maternal genotype determined the timing of birth and influenced fetal lung growth and maturation to ensure perinatal survival [19].

Using the same sets of mouse strains (A/J and B6) [19], the present study was designed to discover genes, pathways, and associated transcriptional networks underlying lung maturation in the two strains of mice that differ in gestational length. Taking into account the dynamic nature of the transcriptional programs accompanying lung maturation, time dependent genome wide lung mRNA profiling changes were assessed using B6 and A/J mice, seeking to identify key regulators and bioprocesses associated with and controlling lung maturation.

## Results

Time course microarray experiments were designed to identify strain dependent and gestation time dependent dynamic changes in lung mRNAs during lung maturation. Lung samples from each mouse strain were collected daily from E15.5 to PN0 at precise gestational ages in the Jackson Laboratory [6]. Lung RNAs isolated from the two mouse strains at different development time points were hybridized to Mouse Gene 1.0 ST Array (n = 3/strain/time). The data have been submitted to Gene Expression Omnibus, Accession No. GSE35485. Principle component analysis of all samples from both strains showed that the primary component (contributes 47% of sample variance) was best correlated with gestational age and the secondary component (contributes 23% of sample variance) correlated with strain. Dynamic lung mRNA expression profiling from B6 and A/J mice were compared to identify: 1) genes and bioprocesses commonly altered in both strains during lung maturation, 2) transcription factors and signaling molecules (TF/SMs) that changed at different stages of lung maturation, 3) pathways and transcriptional networks controlling lung maturation, and 4) strain dependent effects on lung maturation.

### Identification of temporal-dependent gene expression changes during lung maturation

**Figure 1:**
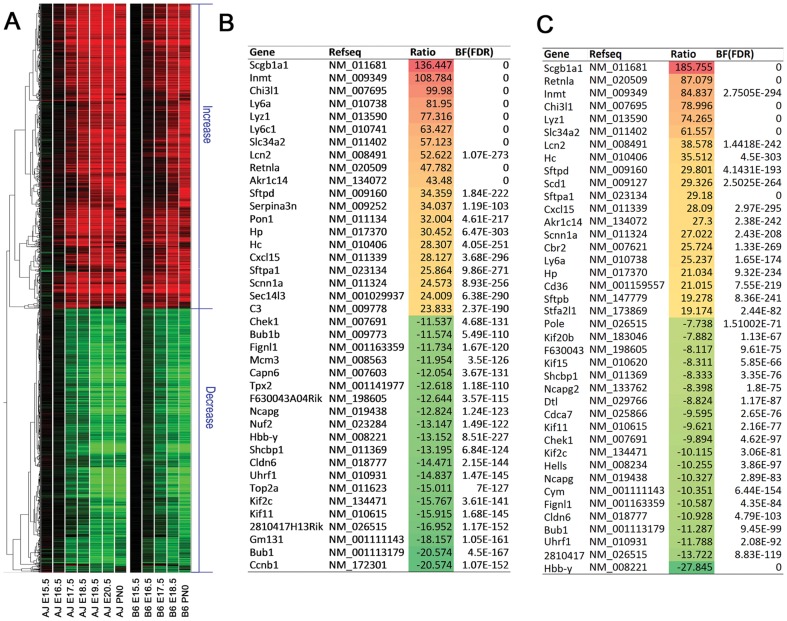
Dynamic changes in lung mRNAs with advancing gestational age. (A) Heatmap of the 1938 mRNAs that were similarly altered in lung from both C57BL/6J (B6) and A/J mouse strains (E15.5 – PN0). Samples were normalized using the mean value of three E15.5 mice from each strain as the baseline. The intensity in red-to-green color indicates the up and down regulation of mRNAs, respectively. Each row represents a single gene, and each column represents a developmental time point for A/J or B6 mice. For each time point, samples from three biological replicates were averaged. (B) and (C) represent the top 20 induced and repressed genes during lung maturation in A/J and B6 mice respectively. The full descriptions for gene symbols can be found under the section of “Abbreviations”.

A functional Bayesian approach [20] was used to analyze time dependent changes in lung mRNAs from both mouse strains. mRNA expression at E15.5 from each strain was used as the baseline. Data from two strains were analyzed separately and then compared to identify genes and bioprocesses commonly altered in both strains. A heatmap of the 1938 mRNAs that were similarly altered in both mouse strains during lung maturation processes is shown in Figure 1A. The top 20 up- and down-regulated genes during lung maturation in A/J and B6 mice are listed in Figures 1B–1C, respectively. Genes involved in innate immune response, fluid/ion transport and surfactant homeostasis were among the most induced on the gene list.

Quantitative RT-PCR was used to validate the mRNA expression of selected lung maturation gene markers. Figure S1 shows the direct comparison of microarray and RT-PCR data on mRNAs at different gestational ages in both A/J and B6 mice. Although the magnitude of transcript expression varied between microarray and RT-PCR, dynamic expression patterns correlated well between the two methods. In general, variability was less for the microarray than for the RT-PCR analysis and differences in mRNA expression determined by RT-PCR analysis were greater than those assessed by microarray analysis for the same genes. The difference is likely related to data normalization.

### Dynamic regulation of transcription factors and signaling molecules during lung maturation

**Figure 2:**
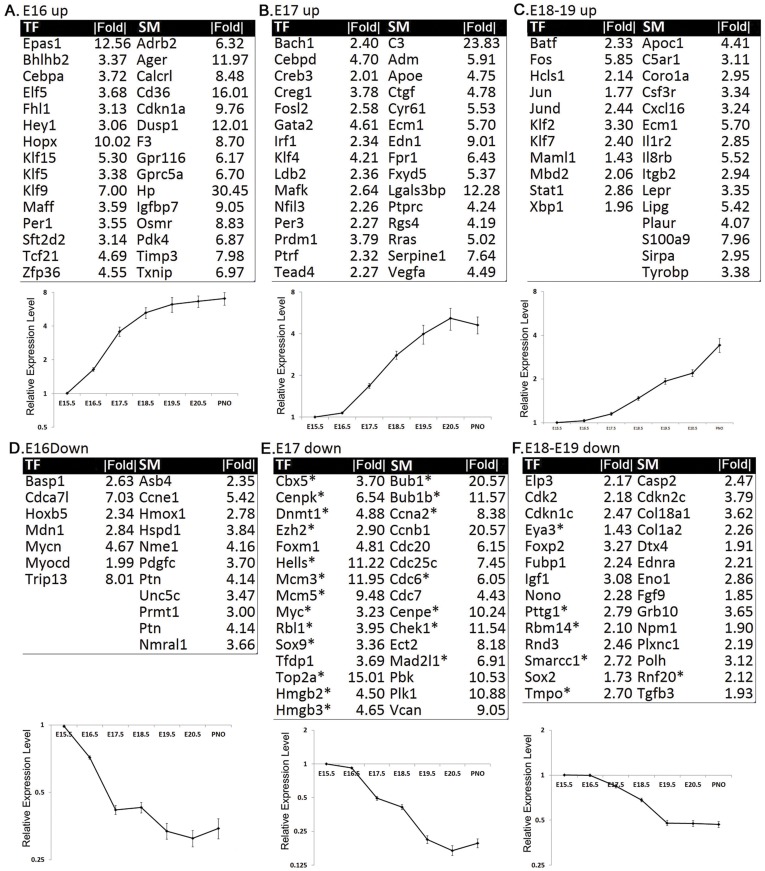
Most changed transcription factors (TF) and signaling molecules (SM) and their dynamic expression profiles at different gestational stages. TF/SMs changed during lung maturation were identified by two-way ANOVA with a cut-off of p-value<0.001 and fold change >1.25. Altered TF/SMs were clustered on the basis of their initial change occurring at E16.5, E17.5, and E18.5 or later, labeled as A) E16 up, B) E17 up, C) E18–19 up, D) E16 down, E) E17 down and F) E18–19 down respectively. Most of the TF/SMs show similar dynamic expression profiles in A/J vs. B6 mouse strains. The x-axis represents the gestational ages and the y-axis (in log_2_ scale) represents relative mRNA using E15.5 as the baseline. |Fold| represents the absolute value of fold change between the peak expression of a given gene (E15.5 – PN0) and its baseline expression at E15.5. Profiles are expressed as the means ± SEM of 3 animals per time point. The full descriptions for gene symbols can be found under the section of “Abbreviations”.

Transcription factors and signaling molecules (TF/SMs) that changed similarly in both mouse strains during lung maturation were identified by two-way ANOVA with a cutoff of p-value<0.001 and fold change >1.25. We chose to use a lower fold change cutoff for TF/SMs since small changes in levels of regulators may mediate large and important downstream effects. We further narrowed down the TF/SMs candidates based on their abundant expression in lung. To assess the abundance of expression of these TF/SMs in the lung, we analyzed their relative mRNA expression in normal adult human lung (using microarray samples from GEO Datasets: GSE16538, http://www.ncbi.nlm.nih.gov/geo/query/), as well as their relative expression in E11.5 mouse lung (GSE9312) [21], [22]. Since the purpose here is to assess the relative expression level in normal lung, only control samples from both datasets were used for intensity distribution analysis. 573 TF/SMs were selected with signal intensity above the 75th percentile in E11.5 mouse lung and/or above the 90th percentile in adult human lung. These selected TF/SMs were enriched in lung tissue and were differentially expressed during lung maturation in both B6 and A/J mice. The selected TF/SMs were clustered using Fuzzy K-means clustering approach [23]. Clusters were further defined by initial changes in their expression at E16.5, E17.5, and E18.5 or later. Representative TF/SMs at different gestational stages and their dynamic changes are shown in Figure 2. The enriched functional categories and known biological associations of TF/SMs that changed at different gestational ages were analyzed using Ingenuity Pathway Analysis tool (IPA).

### Important regulators of lung development are induced at E16

The cluster of TF/SMs induced from E16.5 (Figure 2A) was functionally enriched in the regulation of “cell proliferation” and “lung development”, Figure S2. *Epas1* (endothelial PAS domain protein 1, also known as Hif2a) and *Hopx* (Homeodomain only protein X) were the two most induced TFs in this group, changing 13 and 10 fold respectively, during lung maturation. *Epas1* mRNA is highly expressed in the lung and is known to regulate VEGFA (vascular endothelial growth factor A) expression, blood vessel development and fetal lung maturation [24]. *Hopx* was originally identified in the developing heart where it functions downstream of NKX2.5 (NK2 homeobox 5) to regulate cardiac myocyte maturation in the perinatal period [25], [26]. In the lung, *Hopx* is directly activated by NKX2.1 and GATA6. Activated HOPX, in turn, inhibits NKX2.1 and GATA6, providing a potential negative feedback loop to regulate expression of surfactant associated genes in the lung epithelium. Loss of *Hopx* impaired normal pulmonary maturation, leading to respiratory failure at birth [27]. Other TF/SMs in this cluster known to play important roles in lung development and respiratory epithelial differentiation, including *Cebpa*, *Elf5* (E74-like factor 5), *Klf5* (Kruppel-like factor 5), *Tcf21* (transcription factor 21), *Fgf1* (fibroblast growth factor 1) and *Fgf7* (fibroblast growth factor 7), were induced similarly at this stage of development (Figure 2A, Figure S2).

A small subset of TF/SMs was induced at E16.5, peaked at E17.5, and decreased thereafter. mRNAs in this group included *Fgfr2* (fibroblast growth factor receptor 2), *Lama3* (laminin, alpha 3), *Lama5* (laminin, alpha 5), *Nkx2-1*, *Nr3c1* (nuclear receptor subfamily 3, group C, member 1 (glucocorticoid receptor)) and *Shh* (sonic hedgehog) (Table S1), all known to influence early lung morphogenesis [10], [28], [29], [30], [31], [32], [33]. While the increased expression of this group of TF/SMs likely starts earlier than E15.5, the present experimental design did not include earlier time points needed to fully address the dynamic changes of this subset. Taken together, these data suggest that important regulators of lung development are likely induced at E16.5 or earlier.

### Vegfa signaling is induced at E17

**Figure 3:**
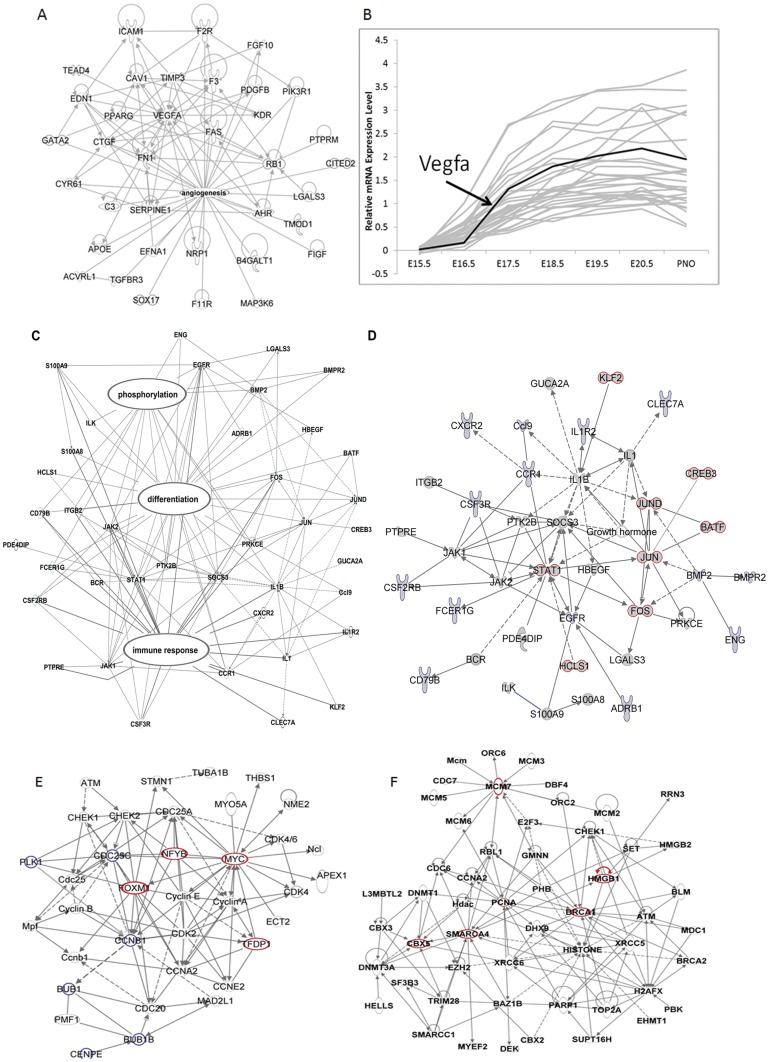
Dynamic changes in TF/SMs during lung maturation. (A) mRNAs encoding TF/SMs that increased from E17.5 were functionally enriched in regulation of angiogenesis. *Vegfa* was identified as an important regulatory hub. (B) mRNAs encoding TF/SMs regulating angiogenesis were highly correlated with *Vegfa* expression (Pearson's correlation coefficients r>0.85). (C) TFSMs induced from E18.5 or later was functionally enriched in genes regulating immune/defense response, cell differentiation and phosphorylation. (D) Biological association networks of TF/SMs indicate that the JAK/STAT1 and AP-1 complexes form important hubs. Cytokine-cytokine receptor interaction was the most enriched signaling pathway at this gestational age. Genes/proteins are represented as nodes, and the biological relationships between two nodes are represented as an edge (line). Solid lines denote direct interactions and dotted lines indirect interactions between nodes. TFs are outlined in red. Cytokines and cytokine receptors are outlined in blue. TF/SMs decreasing from E17.5 to PN0 were functionally enriched in (E) cell cycle and (F) chromatin assembly/organization. In the proposed cell cycle related sub-network (E), *Foxm1*, *Myc*, *Nfyb* and *Tfdp1* formed important TF-hubs (red nodes). The Plk1 signaling pathway was a highly enriched pathway (blue nodes). In the chromatin assembly and histone modification related sub-network (F), the high mobility group family, chromobox family, SWI/SNF family and mini-chromosome maintenance proteins formed important hubs (red nodes).

TF/SMs induced from E17.5 were functionally enriched in mRNAs associated with “cell proliferation” (1.08E-31), “vasculature development/angiogenesis” (7.48E-24), and “apoptosis” (1.91E-20), Figure 2B, Figure S3. Among the TF/SMs regulating vasculature development and angiogenesis, VEGFA forms a central hub (Figure 3A). Other TF/SMs in this group included *Sox17* (SRY-box containing gene 17), *Edn1* (Endothelin 1), *Cyr61* (cysteine-rich, angiogenic inducer, 61), *Tead4* (TEA domain family member 4), *Ahr* (aryl hydrocarbon receptor), *Kdr* (kinase insert domain receptor) and *Nrp1* (neuropilin 1). VEGFA is a critical regulator of angiogenesis and vasculogenesis and plays an essential role in normal lung development and maturation [24]. As shown in Figure 3B, mRNA profiles of this cluster of TF/SMs were positively correlated with *Vegfa* mRNA (r>0.85). Many of the TF/SMs in this group are functionally associated with *Vegfa*, including *Cyr61, Edn1, Tead4 and Nrp1*, all known to induce *Vegfa* expression, and the deletion of *Nrp1 and Cyr61* were embryonic lethal due to vascular defects [34]. The effect of SOX17 in postnatal angiogenesis has been reported [35], but its role in lung vascular development and its relationship with *Vegfa* remain to be determined. The present data support the concept that lung vascular development is carefully timed via the expression of TF/SMs that were largely induced at E17.5.

### TF/SMs regulating “immune/defense” were induced later in gestation

TF/SMs induced at E18.5 or later were highly enriched in mRNAs involved in regulation of immune/defense response (3.77E-13), cell differentiation (6.90E-22) and phosphorylation (5.41E-14). “Cytokine-cytokine receptor interaction” was the most enriched signaling pathway. Enriched functional categories and known biological associations of TF/SM induced in late gestation are shown in Figure 3C–D. STAT1 (signal transducer and activator of transcription 1) and JUN-JUND were identified as important TF hubs in this late gestational gene network. JAK/STAT signaling plays important roles in cytokine-mediated signaling, cell growth, apoptosis and differentiation. Regulators of *Stat1* including JAK-1 and -2 (janus kinase 1,2) and EGFR (epidermal growth factor receptor) were induced in concert with *Stat1* in late gestation. AP-1 (activator protein 1) family members, JUN (jun proto-oncogene), FOS (FBJ murine osteosarcoma viral oncogene homolog) and JUND (Jun proto-oncogene related gene d) form transcriptional complexes implicated in cell differentiation, proliferation, apoptosis, and oncogenesis. AP-1 activity is controlled by the protein kinases ERK1/2 and influenced by other transcription factors including STAT1, BMP-2 (bone morphogenetic protein 2) and TGF-β (transforming growth factor, beta 1) [36]. A model of predicted interactions between the AP-1 and JAK/STAT signaling pathways is shown in Figure 3D. Induction of these pathways in late gestation is likely to influence the maturation of lung innate immunity required to respond to environmental pathogens after birth.

### TF/SMs regulating cell cycle and chromatin assembly decrease during prenatal lung maturation

TF/SMs that clustered on the basis of their coordinate decreased expression during lung development are shown in Figure 2D–F. mRNAs decreasing from E17.5 to PN0 formed the largest cluster (Figure 2E). Networks generated from mRNAs in the “E17 down cluster” were functionally enriched in “chromatin assembly/organization” and “cell cycle”, Figure 3E–F. In the cell cycle related sub-network (Figure 3E), mRNAs in the polo-like kinase 1 (*Plk1*) signaling pathway, including *Plk1*(polo-like kinase 1), *Bub1* (budding uninhibited by benzimidazoles 1), *Bub1b* (budding uninhibited by benzimidazoles 1 homolog, beta), *Ccnb1* (cyclin B1) and *Cdc25c* (cell division cycle 25 homolog C), coordinately decreased from E17.5 to PN0. *Plk1* is a serine/threonine-protein kinase. PLK1-dependent phosphorylation of FOXM1 (forkhead box M1) regulates a transcriptional program essential for mitotic progression [37], [38]. The present data support the concept that decreased PLK1-FOXM1 signaling may mediate the decrease in cell proliferation accompanying prenatal lung maturation. In addition to PLK1-FOXM1 signaling, *Myc* (v-myc myelocytomatosis viral oncogene homolog), *Nfyb* (nuclear transcription factor Y, beta) and *Tfdp1* (transcription factor Dp-1) were also identified as TF-hubs regulating the cell cycle-related network, Figure 3E. TFDP1 heterodimerizes with E2F to enhance DNA-binding activity and promote transcription of E2F target genes [39]. NFYB (nuclear transcription factor Y, beta) binds to c-MYC to regulate cell cycle dependent gene expression [40]. We propose that decreased activity of this network regulates the decreased rate of cell proliferation in the lung with advancing gestation.

Chromatin remodeling and histone modifications have emerged as primary regulatory mechanisms controlling gene expression in eukaryotes. In the present study, mRNAs regulating chromatin structure and organization, including members of the high mobility group family (*Hmga1, Hmgb1, Hmgb2, Hmgb3*), the chromobox family (*Cbx2*, *Cbx3* and *Cbx5*), the SWI/SNF family (*Smrcc1*, *Smarca4* and *Hells*) and the mini-chromosome maintenance proteins (*Mcm2, Mcm3, Mcm5, Mcm6, Mcm7*), decreased from E17.5 to PN0, Figure 3F. HMGB proteins are non-histone chromatin-associated molecules that influence transcription and cell differentiation through histone binding and inhibition of RNA polymerase II activity [41]. The *Cbx* family of genes encodes components of heterochromatin that maintain the transcriptional repressive states of many genes during development via chromatin remodeling and modification of histones [42]. SNF/SWI family members form ATP-dependent chromatin complexes that regulate transcription by altering the chromatin structure at target genes [43]. *Mcm* family members encode components of the MCM2-7 complex essential for DNA replication in eukaryotic cells. Chromatin remodeling through the interaction between the MCM proteins and histone H3/H4 dimers inhibits pol II transcription [44]. TFs in this network are functionally involved in histone modification, including genes influence histone methylation: *Ezh2* (enhancer of zeste homolog 2) and *Ehmt1* (euchromatic histone-lysine N-methyltransferase 1) [45]; histone acetylation & deacetylation: *Smarca4* (SWI/SNF related, matrix associated, actin dependent regulator of chromatin, subfamily a, member 4), *Set* (SET nuclear oncogene) and *Phb* (prohibitin) [46]; and histone phosphorylation: *Baz1b* (bromodomain adjacent to zinc finger domain, 1B) and *Atm* (ataxia telangiectasia mutated) [47]. These TFs are likely to regulate transcription via post-translational modification of histones, to influence the accessibility of TFs to their targets. TFs and enzymes in this network appear to act synergistically to establish local chromatin structure required for control of the cell cycle progressions during lung maturation from E17.5 to PN0.

### Matched dynamic profiles of transcription factors and targets during lung maturation

**Figure 4:**
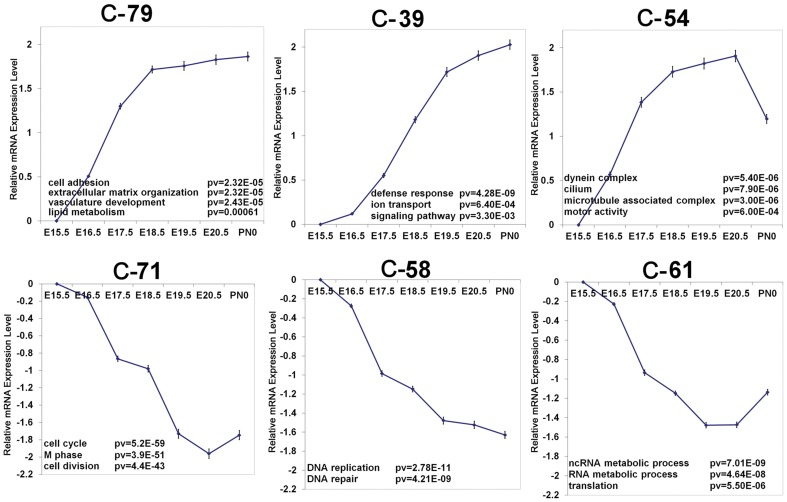
Identification of temporal dependent lung mRNA expression patterns and predicted functions. mRNAs that changed in both strains during lung maturation were clustered into 6 temporally dependent expression patterns using STEM [48]. The x-axis represents the gestational ages and the y-axis represents relative mRNA using E15.5 as the baseline. Profiles are expressed as the means ± SEM of 3 animals per time point. Most highly enriched functional categories for each cluster were identified using DAVID (http://david.abcc.ncifcrf.gov/summary.jsp).

STEM (Short Time-series Expression Miner), a clustering algorithm specifically designed for the analysis of short time series gene expression datasets (5–8 time points) was used to further cluster changes in lung mRNAs with length of gestation [48]. The clustering algorithm first selects a set of representative temporal expression profiles, and assigns each gene to the model profile based on the Pearson correlation. Using STEM, seven representative temporal expression patterns were identified (Figure S4). Among these, clusters 79 and 71 (C-79 and C-71) represent the largest induced or repressed clusters during lung maturation, respectively. Since C-23 was a relatively small cluster (44 genes) and its expression pattern and enriched biological function were similar to C-71, the mRNAs in C-23 were merged into C-71. The clusters and enriched functional classifications are shown in Figure 4. “Cell adhesion”, “extracellular matrix organization”, “vasculature development” and “lipid metabolism” were major bioprocesses that were induced in earlier developmental stages; “defense/immune response”, “ion transport” and “signaling pathway” were identified as major bioprocesses induced later in gestation. mRNAs associated with “Cell cycle”, “DNA repair/replication”, “RNA processing” and “translation” were major bioprocesses that decreased with lung maturation.

Expression patterns of the six temporal dependent clusters were matched with the dynamic expression profiles of TF/SMs using STEM to compute the significance of overlap between the two profiles on the basis of the hypergeometric distribution [48]. Six matched profiles were identified and are shown in Figure S5. Among the 6 matched profiles, C-79 forms the largest cluster induced during lung maturation; 75 TF/SMs share expression profiles with the genes in C-79. C-71 represents the largest “down regulated” group of genes and 54 TF/SM identified from the TF/SMs share expression profiles with gene in C-71.

### Identification of transcriptional networks controlling important bioprocess during lung maturation

**Figure 5:**
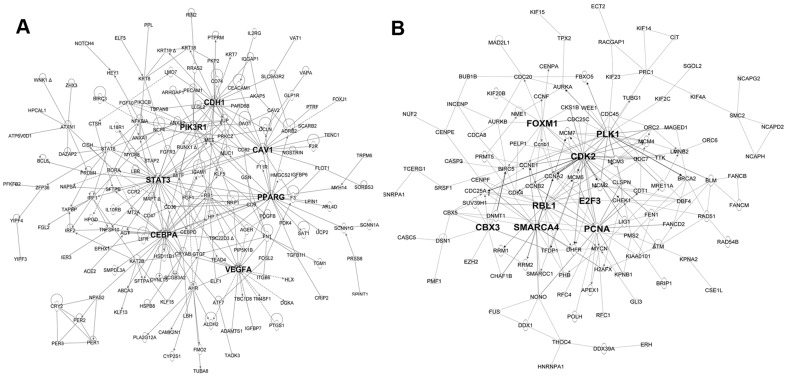
Transcriptional regulatory networks (TRN) of major gene clusters that changed during lung maturation. (A) Proposed TRN for C-79 genes and its matched TF/SMs. (B) Proposed TRN for C-71 genes and its matched TF/SMs. Nodes in bold were TF/SMs that were predicted to serve as important regulatory hubs in the network.

Transcriptional Regulatory Networks (TRN) were generated to reveal the potential biological interrelationships among the matched TF/SMs and their proposed target genes within each cluster. Figure 5A represents the major TRN for genes from C-79 and its matched TF/SMs. PPARG (peroxisome proliferator-activated receptor gamma), CEBPA, VEGFA, STAT3 (signal transducer and activator of transcription 3), PIK3R1 (phosphoinositide-3-kinase, regulatory subunit 1 alpha), CDH1 (cadherin 1) and CAV1 (caveolin 1) were identified as major hubs regulating C-79 genes. Nearest neighbors of the important hubs from the C-79 network were identified and the enriched functions of these sub-networks were assessed. A VEGFA centered sub-network is shown in Figure S6 that was enriched for angiogenesis/vascularization (Pv = 2.3E-26). A CEBPA-PPARG sub-network is enriched in mRNAs regulating lipid metabolism/transport category (1.78E-14) and differentiation (8.7E-14) (Figure S7). A CDH1-CAV1 sub-network is enriched in mRNAs regulating cell adhesion (5.7E-12), cell movement (4.3E-10) & tissue development (2.7E-9) (Figure S8). A STAT3-PI3K sub-network is enriched in mRNAs regulating differentiation (2.36E-25), apoptosis (1.38E-20) and cell proliferation (2.65E-21) (Figure S9).

Similarly, biological association networks were constructed for the major “down-regulated” gene clusters. Network analysis of C-71 and its matched TF/SMs are shown in Figure 5B. The most enriched functions of this network include cell cycle (1.9E-55); DNA replication, recombination, and repair (8.7E-29); and chromosome assembly and organization (2.1E-23). Consistent with the previous dynamic TF/SMs pattern analysis, key regulatory hubs of C-71 included genes important in cell cycle control (e.g. *Foxm1*, *E2F3*, *Cdk2* and *Plk1*) and genes involved in chromatin modification, including *Cbx3* (chromobox 3), *Smarca4*, *Pcna* (proliferating cell nuclear antigen) and *Hmgb1* (high mobility group box 1).

### Strain dependent gene expression during lung maturation

Since the length of gestation of B6 and A/J mice differs by 30 hours, we assumed that dynamic changes in gene expression and cellular processes regulating lung maturation would differ in these two mouse strains. A pairwise Pearson correlation was performed from the lung mRNA microarray data. At E15.5 and E16.5, lung mRNAs were closely correlated and were most divergent at E18.5, Figure S10A. B6 mice were born on E19.5, and A/J mice were born on E20.5. Correlation analysis of neighboring time points suggested that B6 mRNAs at E18.5 were most similar to A/J mRNAs at E19.5 and least similar to A/J mRNAs at E18.5, Figure S10B. These findings were consistent with the observation that A/J fetuses that were delivered two days prematurely (at E18.5) failed to expand their lungs and died of respiratory failure soon after birth [19]. When A/J fetuses were delivered one day prematurely (at E19.5), most pups survived (87.4%), a survival rate similar to that for B6 mice born one day prematurely at E18.5 (82.5%) [19]. Together with previous survival data, the pairwise correlation of mRNAs supports the concept that mRNAs differ the most between these two strains at E18.5, but that catch up in A/J mice at E19.5, are likely to be important for lung maturation and function.

**Figure 6:**
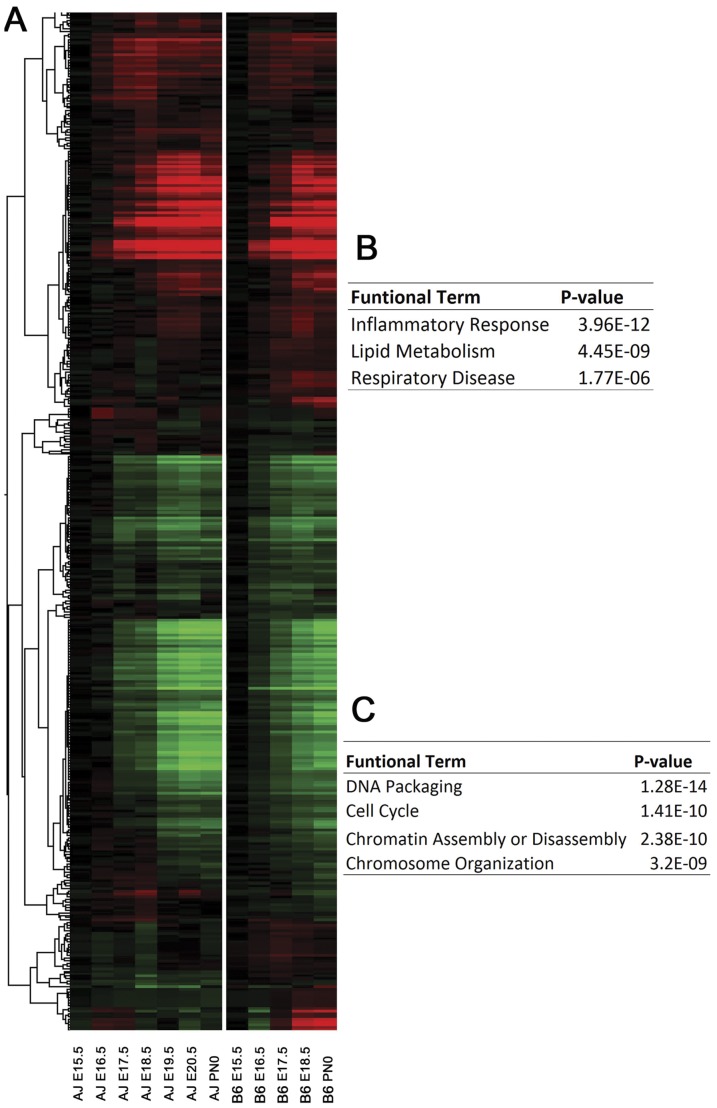
Identification of strain dependent changes in mRNAs during lung maturation. (A) Heatmap of mRNAs expressed differentially in two mouse strains at E18.5 but were not changed comparing A/J at E19.5 to B6 at E18.5. Two major expression patterns were revealed by hierarchical clustering, one is induced with advancing gestational age, and the other is repressed with advancing gestational age. (B) and (C) Analysis of enrichment of functional terms associated with genes in the two clusters. Top functional terms (ranked by p -value) are listed.

We selected genes that were differentially expressed in A/J and B6 mice at E18.5 but unchanged when comparing A/J at E19.5 vs. B6 at E18.5. 390 probe sets corresponding to 304 genes were selected. The heatmap of these strain dependent changes is shown in Figure 6A. Using E15.5 as the baseline, mRNAs of 140 probe sets increased while 250 probe sets decreased during lung maturation. The functional enrichment analysis of mRNAs that increased with gestational age was associated with “Inflammatory/Defense Response”, “Lipid Metabolism” (in particular, regulating phosphatidylcholine) and “Respiratory Disease”. The group of down regulated mRNAs was functionally enriched in “DNA packaging”, “cell cycle”, and “chromatin assembly/disassembly and organization”, Figure 6B–C.

### Genes involved in surfactant homeostasis and innate defense were induced earlier in B6 than in A/J mice

**Figure 7:**
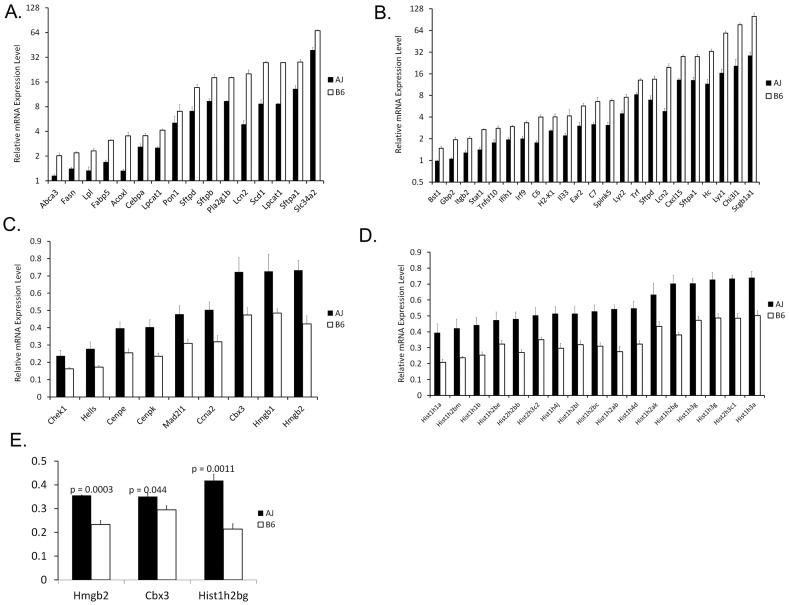
Functional classes of mRNAs associate with strain dependent changes. Genes involved in (A) surfactant homeostasis and (B) innate defense were expressed at significantly higher levels in B6 than in A/J mice at E18.5. (C) Histone associated genes and (D) genes involved in chromatin assembly/disassembly were expressed at significantly lower levels in B6 than in A/J mice at E18.5. (E) RT-PCR validation of selected mRNAs encoding chromatin regulators in lungs from B6 and A/J mice at E18.5 is shown. Results are expressed as the means ± SEM of 3 animals per time point.

Genes regulating pulmonary surfactant are known to be critical for lung function at birth, surfactant deficiency causing RDS in preterm infants [49]. mRNAs encoding surfactant proteins (*Sftpa1*, *Sftpb* and *Sftpd*) and those involved in lipid synthesis (*Fasn*, *Pon1* (paraoxonase 1), *Scd1* and *Lpcat1*), lipid transport (*Abca3*, *Fabp5* and *Slc34a2*) and regulation (*Cebpa*) were increased in B6 compared to A/J mice at E18.5, fold-difference between strains were ranging from 1.5 to 4.1 fold with average fold change of 2.2 (Figure 7A). Mutations in human *SFTPB* (surfactant associated protein B), and *ABCA3* (ATP-binding cassette, sub-family A (ABC1), member 3) genes cause severe lung disease in new-borns, often resulting in respiratory failure at birth [50], [51], [52]. Mutations in *SLC34A2* (solute carrier family 34, member 2), a sodium phosphate co-transporter specifically expressed in type II alveolar cells, cause pulmonary alveolar microlithiasis [53]. Previous studies from this laboratory and others demonstrated that CEPBα is an important regulator of respiratory epithelial differentiation and is required for synthesis of surfactant lipids and proteins necessary for lung function at birth, deletion of the *Cebpa* gene in respiratory epithelial cells in the fetal mouse lung causing respiratory failure at birth [12]. *Lpcat1* (lysophosphatidyl-choline acyltransferase 1) catalyzes the conversion of lysophosphatidylcholine to phosphatidylcholine, which accounts for up to 75% of surfactant lipid saturated phosphatidylcholine (SatPC) synthesis in type 2 cells. *Lpcat1* mRNA and acyltransferase activity correlated directly with SatPC content, surfactant function, and perinatal survival [54].

Genes involved in lung innate immunity (*Sftpa1, Sftpd, Scgb1a1, Il33, Gbp2, Stat1, C3, C6, C7, Ln2, Tnfsf10, Iflh1 and Itgb2*) were expressed at significantly higher levels in lungs of B6 than in A/J mice at E18.5, fold-difference between strains were ranging from 1.5 to 4.1 fold with average fold change of 2.4, Figure 7B. Literature mining indicated that NF-κB is likely a common mediator of these genes regulation during inflammation. SP-A and SP-D, members of the collectin family of lectins, play important roles in the innate immunity in lung, binding to bacterial, fungal, and viral pathogens and facilitating their uptake by alveolar macrophages, and regulating macrophage migration and cytokine production [55]. NF-κB (p65/p50) binds to the *SFTPA1* (surfactant protein A1) promoter, activating its transcription [56]. SP-D (surfactant protein D) regulates NF-κB and matrix metalloproteinase production in alveolar macrophages [57]. Interleukin 33 (IL-33), a member of the IL-1 family of cytokines, enhances production of Th2-associated cytokines through the activation of MAPK (mitogen-activated protein kinase 3) and NF-κB [58]. Neutrophil adhesion to extracellular matrix is necessary for an effective inflammatory response and is mediated by beta2-integrin (Itgb2) that is induced via NF-κB activation [59]. Expression of complement components (C5, 6 and 7) were induced at higher levels in B6 at E18.5. Complement proteins are known to influence innate immune defense, modulate cell survival, growth, and differentiation in various tissues [60]. The stimulation of C3 and C5 receptors leads to NF-κB activation in liver [61]. The prenatal induction of this network of genes associated with NF-κB and control of innate immunity is consistent with the importance of host defense in the lung necessary for survival after birth when the lung is exposed to alveolar pathogens.

The association between prenatal infection, inflammation and lung maturation is well established [62], but the underlying mechanisms integrating these processes are unclear at present. On the basis of the present and previous studies, we propose that NF-κB is likely a central mediator of the accelerated lung maturation seen after infection [63], [64]. Findings that surfactant and inflammatory response/innate defense related lung mRNAs were induced in both mouse strains and expressed earlier in B6 (shorter gestation length), implicate NF-κB as an important signaling mechanism that is likely to influence the timing of lung maturation. Many of the proteins regulating surfactant structure and function play dual roles in the regulation of innate immunity. Taken together, these data support the concept that innate immunity and surfactant production are critical and connected processes that prepare the new born for air breathing at birth.

### Strain dependent regulation of genes controlling chromatin assembly

In contrast to the relatively higher expression of innate defense response and surfactant-related genes during lung development in B6, the expression of genes encoding histones (H1–H4) and other regulators of chromatin assembly/disassembly decreased with advancing gestation and were expressed at significantly lower levels in B6 vs. A/J mice at E18.5, fold-difference between strains were ranging from 1.5 to 2.0 fold with average fold change of 1.62, Figure 7C–E. Chromatin regulators play a key role in generating diversity in gene expression of closely related species and strains [65]. We hypothesize gestational differences in lung mRNAs influencing chromatin organization may contribute to the differences in lung maturation between the A/J and B6 strains. Since many of the genes regulating chromatin assembly function as transcriptional repressors, their earlier decrease in B6 mice may contribute to the shorter time needed for lung maturation in B6 compared to A/J mice.

### Predicted inhibitory role of Notch signaling during lung maturation

**Figure 8:**
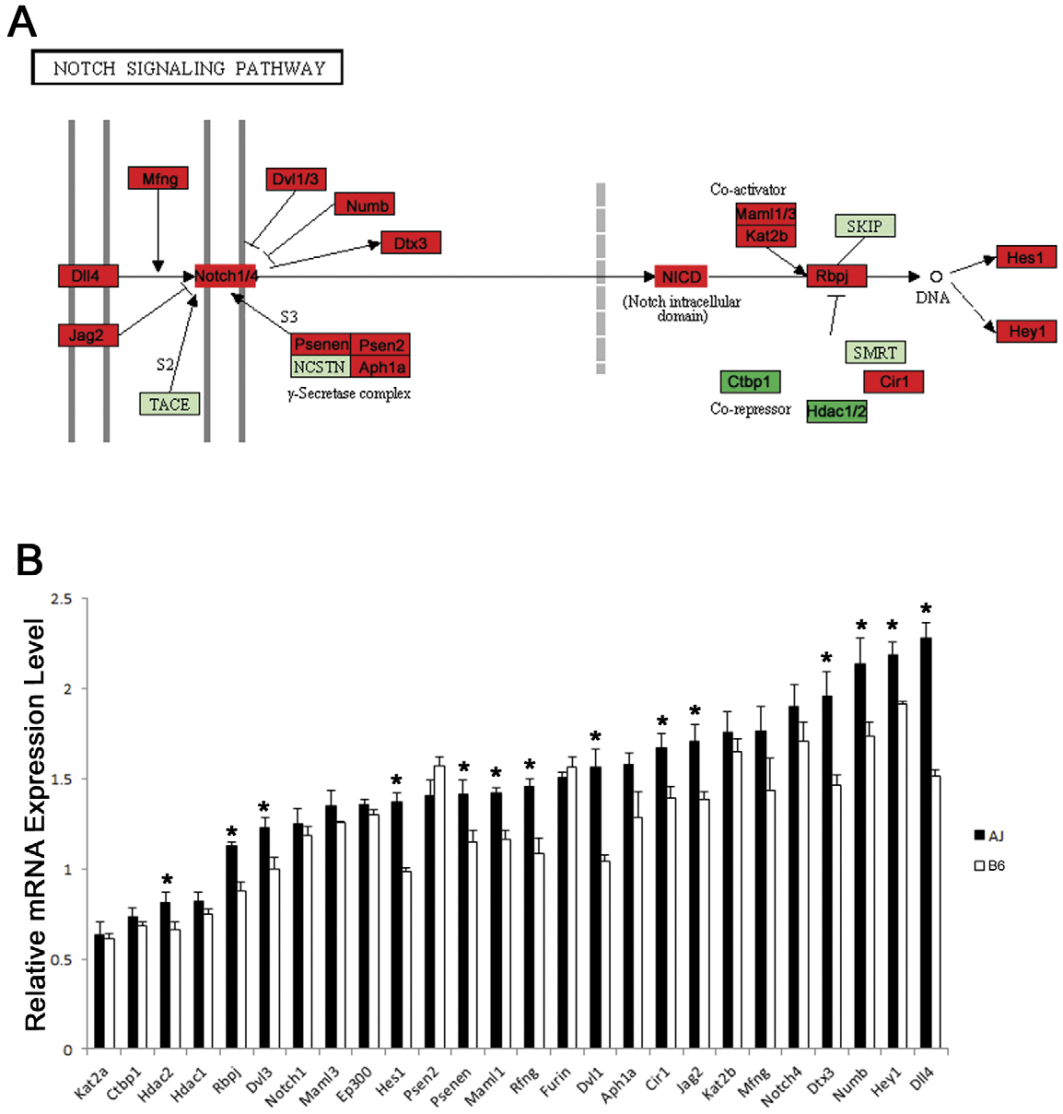
Changes of lung mRNAs related to the Notch Signaling Pathway were influenced by strain. (A) Representation of mRNAs changed during lung maturation in KEGG Notch Signaling Pathway (map04330). Red nodes indicate mRNAs that were significantly increased, while dark green indicates decreased expression during lung maturation. Light green indicates no significant change in gene expression or data not available. (B) Genes encoding a number of key components in the Notch Signaling Pathway were expressed at significantly higher levels in A/J than in B6 mice at E18.5 (*: P<0.05). Results are expressed as the means ± SEM of 3 animals per time point.

Notch signaling is an evolutionarily conserved intercellular signaling pathway essential for embryonic development and the determination of cell fate in multiple organ systems including the lung [66], [67], [68], [69]. Notch signaling is typically initiated by interaction of Notch receptors with ligands, which leads to activation of the transcriptional repressors HES and HEY. As the primary downstream effectors of Notch signaling, HES and HEY family of TFs are considered as central regulators of fate-specific gene transcription [70]. When Notch signaling is activated, *Hes* and *Hey* genes are actively transcribed and cell differentiation is suppressed; whereas when Notch signaling is inactive, cells are allowed to proceed to a specific differentiation fate [67], [71]. In the present study, mRNAs encoding Notch ligands (*Dll4, Jag2*), Notch receptors (*Notch1* and 4), Notch downstream effectors (*Hes1* and *Hey1*), as well as a number of other key components in the Notch pathway were induced in both mouse strains (Figure 8A), indicating that active Notch signaling is associated with lung maturation. Furthermore, in contrast to the relatively higher expression of *Cebpa* (a known positive transcriptional regulator of lung maturation) in B6 vs. A/J mice, the mRNAs encoding *Hes1* (hairy and enhancer of split 1) and *Hey1* (hairy/enhancer-of-split related with YRPW motif 1) and other key components in the Notch signaling were expressed at significantly higher levels in A/J than in B6 mice at E18.5, Figure 8B. Our previous study showed that co-transfection with a plasmid expressing constitutively active Notch in mouse lung epithelial cells repressed the expression of several maturation-associated lung mRNAs, including *Abca3*, *Sftpc* (surfactant protein C), *Lyz1* (lysozyme 1), *Lyz2* (lysozyme 2), and *Lpcat1* [19]. Together, our data suggests a potential inhibitory role for Notch signaling in perinatal lung maturation. We hypothesize that both positive and inhibitory regulators are required to achieve the precise regulation of the inverted relationship between lung cell proliferation and differentiation that changes with advancing gestational age. The net-balance between the inducers and repressors may be critical in determining the timing of maturation difference in A/J and B6 mice.

Although our observation is consistent with the “classical” role of Notch signaling, which is to prevent cell differentiation and to promote cell proliferation, it is important to note that Notch signaling is highly context dependent, such that stage, dosage, and cell-type differences all influence the biological outcome [69], [72], [73]. Future functional data will be needed to clearly define the role of Notch signaling in the maturational process of the many distinct cell types that comprise the lung.

## Discussion

The lung is a complex organ consisting of more than 40 distinct cell types derived from ectodermal, mesenchymal and endodermal compartments with a number of specialized functions related to gas exchange, host defense, and ion transport [5]. Lung development is a highly coordinated process that can be dissected into five morphologically distinct stages that begin near mid-gestation and continue through the early postnatal period. The embryonic stage is distinguished by the formation of the lung buds and division of the tracheal-esophageal tube (E9–11.5). The pseudoglandular stage (E11.5–15.5) is characterized by branching of the conducting airway, formation of the peripheral acinar tubules and buds, and vasculogenesis; the canalicular stage (E15.5–17.5), by expansion of the acinar tubules and buds, angiogenesis, and differentiation of alveolar epithelial type I and II cells; the saccular stage (E17.5 – PN5), by dilation of the terminal respiratory sacs, thinning of the mesenchyme, and deposition of elastin; and the alveolar stage (PN5-30), by maturation of the alveolar-capillary bed, alveolar ducts and alveoli [74], [75]. Peripheral lung maturation begins at approximately E15.5 and increases dramatically prior to birth. Differentiation of distal respiratory epithelial cells is required to establish effective gas exchange at birth, as well as increased production of pulmonary surfactant lipids and proteins that are essential for lung function and host defense after birth. Historically, concepts of lung “maturation” were focused primarily on the induction of surfactant lipids and proteins that are deficient in RDS found in preterm infants. A detailed understanding of the biological processes governing stage specific changes during lung maturation remain incompletely defined, but will be useful in designing new diagnostic and therapeutic strategies for this common clinical disease.

**Figure 9:**
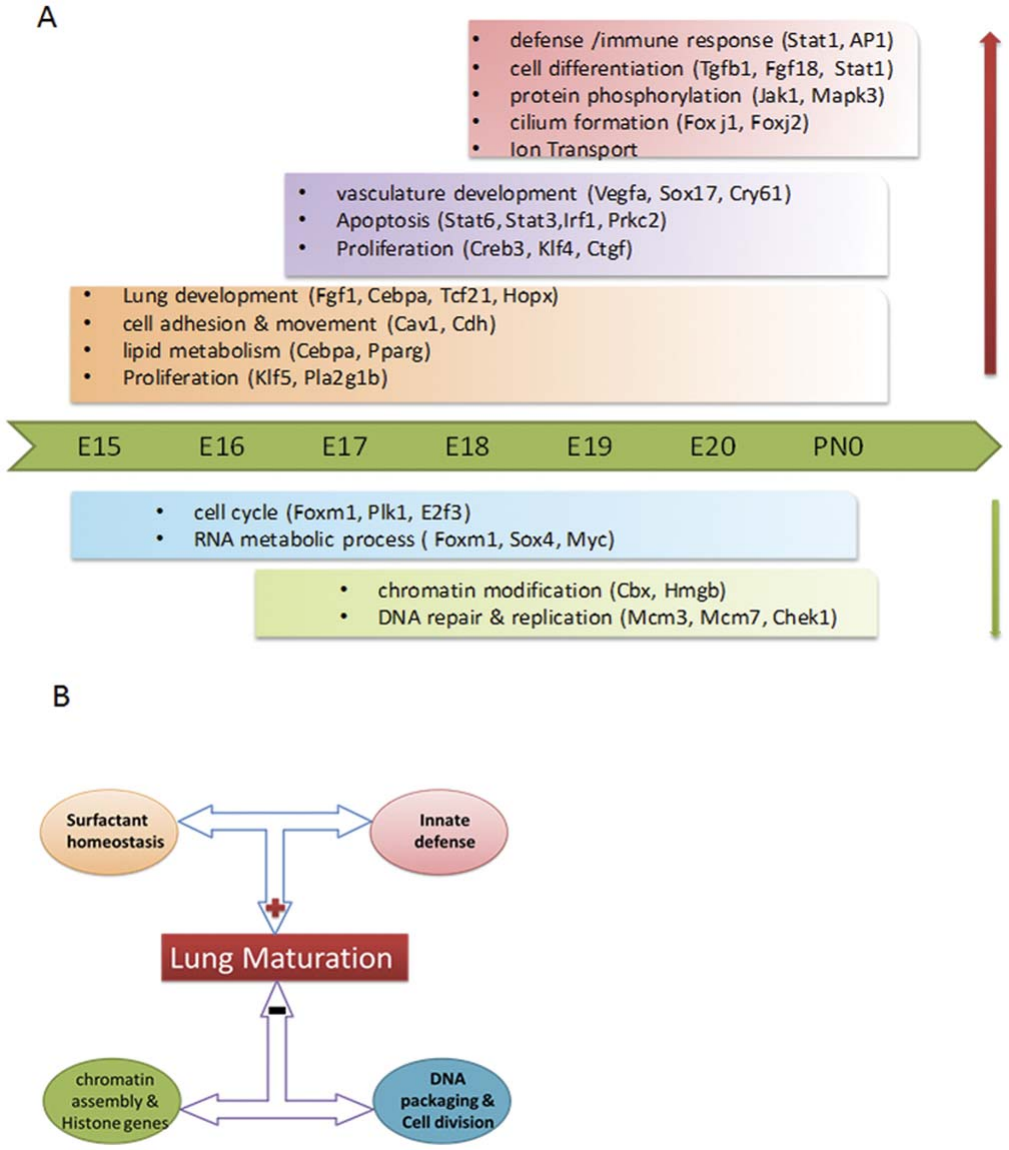
Schematized depiction of temporal and strain dependent effects on lung maturation. (A) Representation of bioprocesses and predicted key regulators changed dynamically with advancing gestation. (B) Strain effects underlying lung maturation are shown. Innate immune responses and surfactant production are critical and connected processes that play positive roles to promote lung maturation, while epigenetic regulators are likely to play a repressive role by altering chromatin structure and controlling the cell cycle. The precise balance of these positive and negative gene networks is likely a critical determinant that coordinates the timing of lung maturation.

Genome-wide, time-course studies are a powerful tool for exploration of mechanisms underlying biological processes in many areas of biomedical research. Using this strategy, we systemically explored the dynamic regulation of lung maturation in both B6 and A/J mouse strains and identified and experimentally verified a number of genes, processes, pathways and transcription networks associated with, and likely to regulate, the complex processes of prenatal lung maturation. Both temporal and strain dependent gene expression patterns were identified during lung maturation. As illustrated in Figure 9, bioprocesses and key regulators associated with different stages of lung development were identified. Lung development, cell adhesion and movement, lipid metabolism, and proliferation were induced early in lung maturation (E15–16, pseudoglandular stage). *Hopx*, *Cebpa*, *Tcf21* and *Klf5* were predicted to be important transcriptional regulators at this stage, a finding consistent with gene deletion studies that support their important roles in prenatal lung maturation [12], [27], [76], [77]. TF/SMs regulating vasculature development and apoptosis were induced at E16–17 (canalicular stage), *Vegfa, Sox17 and Stat3/6* representing important regulators at this stage. Innate defense/immune responses, cell differentiation, protein phosphorylation, ion transport, and cilium formation were induced at later gestational ages (E18–20, in the saccular stage), *Stat1, Tgfb1* (transforming growth factor, beta 1) *and Foxj1* (forkhead box J1) being important regulators associated with this stage of maturation. Cell cycle and chromatin assembly were repressed during lung maturation. FOXM1, PLK1, chromobox, SWI/SNF and high mobility group families of transcription factors were predicted to play important roles in the negative regulation of lung cell proliferation occur in late gestation. The structure of the mammalian lung is highly conserved among mammals [78]. The morphogenetic processes of alveolarization and septal maturation across species are similar, but the timing of lung maturation and the degree of lung maturity at birth varies considerably among species, reflecting their gestational lengths [79], [80]. Since a rich capillary network and close apposition of endothelial cells and alveolar epithelial cells must be present for efficient gas exchange, the structural maturation of the lung at birth varies from the late canalicular to early terminal sac stage [81]. Mechanisms controlling the timing of lung maturation remain unclear. The present study was designed using inbred mouse strains that vary in gestational length, providing comprehensive information regarding strain dependent differences that underlie the timing of lung maturation prior to birth. Present findings demonstrate that prior to birth, innate immune responses and surfactant production are critical and connected processes that positively influence lung maturation necessary for respiration and survival after birth. In contrast, epigenetic regulators are likely to play a repressive role by altering chromatin structure and controlling the cell cycle (Figure 9B). We hypothesize that precise regulation and balance among the positive and negative gene networks are likely critical determinants coordinating the timing of lung maturation with gestational length that differs in the B6 and A/J mouse strains.

Three sets of lung developmental time course microarray studies have been published previously with the emphasis on dynamic changes in expression of genes influencing extracellular matrix, tumorigenesis and miRNA/mRNA interactions respectively [82], [83], [84]. Kho et al. [85] combined the first two sets of array data and re-analyzed the data by principle components analysis (PCA). Through PCA analysis, these investigators identified a dominant component that was closely correlated with the distance from sample age to day of birth. Genes contributing to this pattern were functionally enriched in “oxygen and gas exchange” and “host defense”. Cox et al. [86] compared proteomic profiles with parallel DNA microarray data, identifying significant correlations and differences between protein and transcript levels during lung development. Although the overall correlation was relatively low (643 of the 1383 protein microarray data pairs were positively correlated), protein/mRNA pairs correlated with decreasing expression were enriched in genes associated with DNA replication, chromosome organization and biogenesis, findings consistent with our data, supporting the concept that repression of cell cycle and chromatin organization genes play important roles in prenatal lung maturation. Dong et al. [83] compared lung miRNA and mRNA profiling from E12 to adulthood to identify dynamically regulated miRNAs, their genome localization, and likely targets. The major Bioprocess patterns identified from previous mRNA profiling analysis [83] were largely consistent with our analytic results.

Our study differs from previous studies in many aspects, including experimental design, analytic approaches and Chip platform. The present study was uniquely designed to address strain dependent changes in lung maturation and to explain relationships between gestation length and lung maturation prior to birth. Previous studies adapted methods, such as T-test, ANOVA, K-means and hierarchical clustering, or Principle components analysis, to identify differentially expressed genes and cluster analysis. These methods were designed for static experiments that do not take into account temporal relationships among samples, thus, losing sequential information provided by the time series. We applied a functional Bayesian approach BATS (Bayesian Analysis of Time Series) in which each gene expression temporal profile was estimated globally by expanding it on an orthogonal basis [20]. This Bayesian approach is suitable for handling small sample numbers, irregular sampling intervals, missing data, and genes' temporal dependence between observations. For comparison purposes, we also conducted data analysis using traditional methods, such as two-way ANOVA and EDGE (Extraction of Differential Gene Expression), the first functional approach proposed for microarray time-course analysis [87]. The resulting gene list from BATS displayed improved temporal dependent patterns and reduced noise. For cluster analysis, we chose to use Short Time-series Expression Miner [48] for temporal dependent pattern recognition and selected only statistically significant patterns for further functional analysis. Previously published studies were performed using Affymetrix Mu11K and U74Av2 Chip platforms (contains 7202 and 8809 genes respectively, with 6667 genes in common), but we used Mouse Gene 1.0 ST Array, the latest product for Affymetrix expression arrays, which has complete genome coverage (total of 28853 genes), improved sensitivity, specificity, and reproducibility.

With these unique experimental designs and methodologies in hand, the present study revealed important insights into the gene networks and associated molecular and cellular mechanisms underlying the complex changes in lung structure and function that is broadly termed “lung maturation”. A number of limitations to the current study should be noted. One is that the design only covers the periods from middle to late gestational ages (E15.5 to PN0) that were focused primarily on prenatal lung maturation and, thus, may have missed important transcription factors and signaling pathways regulating lung formation earlier in development. Previously published studies used lung samples from E12 to adulthood [83], [85]. Therefore these earlier studies serve as a useful extension of data that are of interest for the study of lung biology. Another limitation is that so far all microarray studies derived from whole lung samples and, thus, does not represent dynamic changes of mRNAs in different populations of distinct lung cell types in the developing lung. Another important limitation of the present approach is that the correlation between regulators and potential targets are largely based on their shared expression patterns, which is likely to miss important TFs that regulate their targets independently of their own levels of expression. The likelihood of these influences is indicated by the apparent lack of identification of TFs, including *Foxa2, Etv5* (ets variant 5) *and Nfatc3*, which are known to regulate genes important for of lung maturation. While expression of their target genes increased, the relative abundance of these TFs did not change substantially with advancing gestation, supporting the concept that their activity is not mediated primarily at transcript levels but perhaps by post-transcriptional mechanisms. The integrative approach we previously developed can be used as an important extension to the current study [16]. In that study, we predicted the TF-Target matching pairs that regulate surfactant homeostasis by integrating analytic results from gene expression profiling, protein interaction, functional annotation, and promoter and literature mining and there we identified cis-elements, including HNF3B, NFAT and ETSF, as major regulatory hubs controlling surfactant homeostasis [16].

In summary, the present study systemically mapped key regulators, bioprocesses and transcriptional networks controlling lung maturation, providing new insights into the molecular and cellular mechanisms underlying lung maturation and function. A thorough understanding of the molecular mechanisms controlling the timing of normal lung maturation processes will promote the understanding of the pathogeneses of lung diseases associated with preterm birth, providing new potential therapeutic and diagnostic tools to treat pulmonary diseases in infants.

## Materials and Methods

### Microarray Design and Lung RNA Isolation

Mice were maintained in a pathogen-free environment in accordance with protocols approved by the Jackson Laboratory Animal Care and Use Committee. RNA analysis of murine tissue was approved by the CCHMC Institutional Animal Care and Use Committee. All microarray data are MIAME compliant and submitted to GEO (http://www.ncbi.nlm.nih.gov/geo/). All lung samples were carefully collected at precise gestational and postnatal ages, determined by continuous video-monitoring of mating and delivery times in the Jackson Laboratory [18]. Lungs from 3 litters (3 males/litter as one pool) of each strain were used at each gestational and postnatal time point (E15.5, E16.5, E17.5, E18.5 and PN0 for B6; E15.5, E16.5, E17.5, E18.5, E19.5, E20.5 and PN0 for A/J). The interval between all-time points was 24 hours, except for E20.5 (just prior to birth) and PN0 (just after birth) for the A/J strain, for which the perinatal interval was 3 to 6 hours. RNA was isolated from whole right lung homogenates using an RNAeasy Protect mini kit (Qiagen, Valencia, CA). Lung RNA isolated from two mouse strains at different development time points (n = 3/strain/time) were hybridized to the Mouse Gene 1.0 ST Array, the latest product in the family of Affymetrix expression arrays offering whole-transcript coverage (Affymetrix, Santa Clara, CA). The RNA quality and quantity assessment, probe preparation, labeling, hybridization and image scan were carried out in the CCHMC Affymetrix Core using standard procedures. The complete dataset has been submitted to the Gene Expression Omnibus (GEO) database with the accession number of GSE35485.

### RNA Microarray Analysis

mRNA array data was normalized using the Robust Multichip Average model [88], [89] and analyzed using three different statistical methods, including Bayesian Analysis of Time Series (BATS) [20], Extraction of Differential Gene Expression (EDGE) [90], and two-way ANOVA. The results were compared using Venn diagrams and hierarchical clustering for visualization. We chose to use BATS results for genes that were dynamically changing during lung maturation, since gene lists from BATS displayed clear temporal dependent patterns with reduced noise. Using this approach, gestational time dependent, differential gene expression profiles were identified from both B6 and A/J time series. Genes differentially expressed during lung maturation were selected with the threshold of Bayes Factor (similar to false discovery rate [91]) ≤0.01 and fold change (defined as the ratio of the maximal and the minimal expression levels in all time points of a given gene) ≥2.0 or ≤0.5. The overlapping genes were commonly altered in both mouse strains along with the lung maturation processes.

Pearson correlation was performed among all samples to estimate the sample similarity and divergence between two strains at different gestational ages. Strain-dependent, differential, gene expression profiles were identified between B6 and A/J time series first using two-way ANOVA with strain and time as two independent variables and time-strain interaction as the dependent variable. Genes differentially expressed between the strains were selected with a threshold of p-value (Strain) <0.0001 and a fold change (defined as the ratio of the maximal and the minimal expression levels in all time points of a given gene) of either ≥2.0 or ≤0.5. To identify strain dependent changes prior to birth, we compared mRNA expression in A/J vs. B6 mice at E18.5 and A/J at E19.5 vs. B6 at E18.5. We selected genes that were differentially expressed in A/J and B6 mice at E18.5 but unchanged when comparing A/J at E19.5 vs. B6 at E18.5.

### Identification of temporal dependent gene expression patterns

Differentially expressed mRNAs varying with advancing gestation were further clustered into distinct subgroups using Short Time-series Expression Miner (STEM) [48]. STEM is a unique clustering algorithm specifically designed for the analysis of short time series microarray gene expression data that is implemented in four steps: 1) selecting model profiles; 2) assigning genes to model profiles; 3) identifying significant model profiles; and 4) grouping significant profiles based on similarity. Using this method, temporal expression patterns of mRNA were identified with a permutation test p-value≤0.05.

### Functional classification and pathway analysis

Time and strain dependent changes in lung mRNAs were subject to gene ontology and pathway analysis. Gene Ontology Analysis was performed using public available web-based tool David (database for annotation, visualization, and integrated discovery) [92]. Over-represented pathways were identified by comparing the overlap of differentially expressed genes and all genes in the mouse genome with the gene sets associated with known pathways and gene sets from Ingenuity knowledge base (Ingenuity Systems, Redwood City, CA), MSigDB (http://www.broadinstitute.org/gsea/msigdb/), KEGG (http://www.genome.ad.jp/kegg/), Reactome (http://www.reactome.org/) and Biocarta (http://www.biocarta.com/). A pathway was considered to be over-represented when a probability p-value≤0.01 and gene hits ≥5 were found.

### Matched dynamic profiles of transcription factors and their potential targets

Transcription factors and signaling molecules (TF/SMs) that changed during lung maturation were identified using two-way ANOVA (time) with the p-value≤0.0001 and fold change ≥1.25. The dynamic expression patterns of the transcription factors/signaling molecules were compared with the patterns of the differentially expressed genes identified by the functional Bayesian models using data from both strains to link TF/SMs to potential target genes based on their expression similarities. Candidate TF/SMs associated with a particular expression pattern were further filtered using a promoter common elements search (Genomatix). If a TF displayed a similar dynamic expression pattern with a gene cluster and the corresponding TF binding sites was enriched in the promoters of that gene cluster, the TF was considered a potential regulator of that cluster of genes. Signaling molecules sharing expression or functional similarity with the matched TFs were considered to be potential upstream regulators connected with the TFs and targets genes in the same cluster. The regulatory relationships were analyzed using Ingenuity Pathway Analysis (IPA, Ingenuity Systems, Redwood City, CA). IPA software maps the transcription factors, signaling molecules, and cluster genes identified from the microarray and promoter analysis onto the interactome according to Ingenuity Pathway Knowledge Base, a large curated database of published literature findings on mammalian biology. Genetic networks preferentially enriched by input genes were generated based on their connectivity. Statistical scores were then calculated to rank the resulting networks and pathways using Fisher's right tailed exact test. The score indicates the degree of relevance of a network to the input gene set, relative to the number of network-eligible genes and the size of the network.

### RT-PCR Validation

RNA was isolated from whole right lung homogenates using an RNAeasy Protect mini kit (Qiagen, Valencia, CA). Three mice were pooled for each sample and four samples were used for each gene (n = 12 mice per group) and each sample was run in triplicate. Quantitative RT-PCR was analyzed using TaqMan® gene expression assays (Applied Biosystems, Foster City, CA). Probe and primer sets for 18s rRNA were used for normalization. All probes were selected from the list of Applied Biosystems. Quantitative RT-PCRs were performed with TaqMan primers listed in Table S2.

## Supporting Information

Figure S1 **Validation of lung mRNAs of selected genes.** The x-axis representing lung samples obtained at each gestational age. The left y-axis is log2 transformed relative mRNA levels determined by QRT-PCR from previous study. The right y-axis is log2 transformed relative mRNA levels determined by mRNA microarray. Red and blue lines represent the expression profiles determined by microarray analysis and QRT-PCR respectively.
(TIF)

Figure S2 **TF/SMs induced from E16.5 were functionally enriched in regulation of cell proliferation and organ development (in particularly lung development).** TF/SMs changed during lung maturation were identified by two-way ANOVA and clustered on the basis of their initial change occurring at E16.5, E17.5, and E18.5 or later. The enriched functional categories of TF/SMs induced at different gestation ages were analyzed using Ingenuity pathway Analysis tool (IPA).
(TIF)

Figure S3 **TF/SMs induced from E17.5 were functionally enriched in the regulation of cell proliferation, vasculature development/angiogenesis and apoptosis.** TF/SMs changed during lung maturation were identified by two-way ANOVA and clustered on the basis of their initial change occurring at E16.5, E17.5, and E18.5 or later. The enriched functional categories of TF/SMs induced at different gestation ages were analyzed using Ingenuity pathway Analysis tool (IPA).
(TIF)

Figure S4 **Genes commonly changed during lung maturation were clustered into 7 temporal dependent expression patterns using STEM.**
(TIF)

Figure S5 **Dynamic expression profiles match of TFSMs and their target genes during lung maturation.** We used temporal dependent genes commonly altered in both mouse strains during lung maturation as original set profiles (profiles in the left panel) and TFSMs dynamically changed during lung maturation as comparison set profiles (profiles in the right panel). Significant pattern matches were identified by STEM.
(TIF)

Figure S6 **A VEGFA centered sub-network is functionally enriched in mTNAs involved in angiogenesis/vascularization.** Yellow nodes are known binding partners of VEGFA. Orange nodes are known to regulate VEGFA expression and green nodes are genes regulated by VEGFA.
(TIF)

Figure S7 **CEBPA-PPARG sub-network regulates lipid metabolism/transport and cell differentiation.** Dynamic expression patterns of developmentally changed genes and TF/SMs were matched using STEM. Nearest neighbors of the important hubs from the C79 network were identified and the biological associations of genes in these sub-networks were assessed using Ingenuity pathway Analysis tool (IPA).
(TIF)

Figure S8 **CDH1-CAV1 sub-network regulates cell adhesion, cell movement & tissue development.** Dynamic expression patterns of developmentally changed genes and TF/SMs were matched using STEM. Nearest neighbors of the important hubs from the C79 network were identified and the biological associations of genes in these sub-networks were assessed using Ingenuity pathway Analysis tool (IPA).
(TIF)

Figure S9 **STAT3-PI3K sub-network regulates differentiation, apoptosis and cell proliferation.** Dynamic expression patterns of developmentally changed genes and TF/SMs were matched using STEM. Nearest neighbors of the important hubs from the C79 network were identified and the biological associations of genes in these sub-networks were assessed using Ingenuity pathway Analysis tool (IPA).
(TIF)

Figure S10 **Close correlation of lung mRNAs from B6 (E18.5) and A/J (E19.5).** (A) Pearson correlation of lung mRNAs of B6 and A/J mice from E15 to PN0. Each sample was labeled to indicate the strain, age and biological replicates number of the mouse (i.e., AJ E15.1 represents the lung sample from AJ mouse strain at age of E15, litter number 1) (B) Scatterplot of correlation matrix on E18.5 and its neighboring time points. Correlation matrix was generated using multivariate analysis in JMP 9.0 (SAS Institute, Inc. Cary, NC).
(TIF)

Table S1 **TFSMs induced from E16.5, peak at E17.5 and decreased thereafter.** The dominant expression pattern for TFSMs induced from E16.5 was shown in Figure 2A (i.e., consisting a fast increasing phase from E15–E18 and a slow increasing phase from E18-PN0). There is a small subset of TFSMs induced from E16.5, but peaked at E17.5 and decreased thereafter; genes in this group were known to influence early lung morphogenesis.
(DOCX)

Table S2 **Taqman primers used for quantitative RTPCR.** Taqman primers (Applied Biosystems Catalog No.) were selected on the basis of microarray experiments examining mouse lung transcript levels during lung maturation.
(DOCX)
