# Supplementary figures and images for: Transcriptional Programs Controlling Perinatal Lung Maturation
Source: PLoS One. 2012 Aug 20;7(8):e37046. doi: 10.1371/journal.pone.0037046 (PMC3423373; doi:10.1371/journal.pone.0037046)

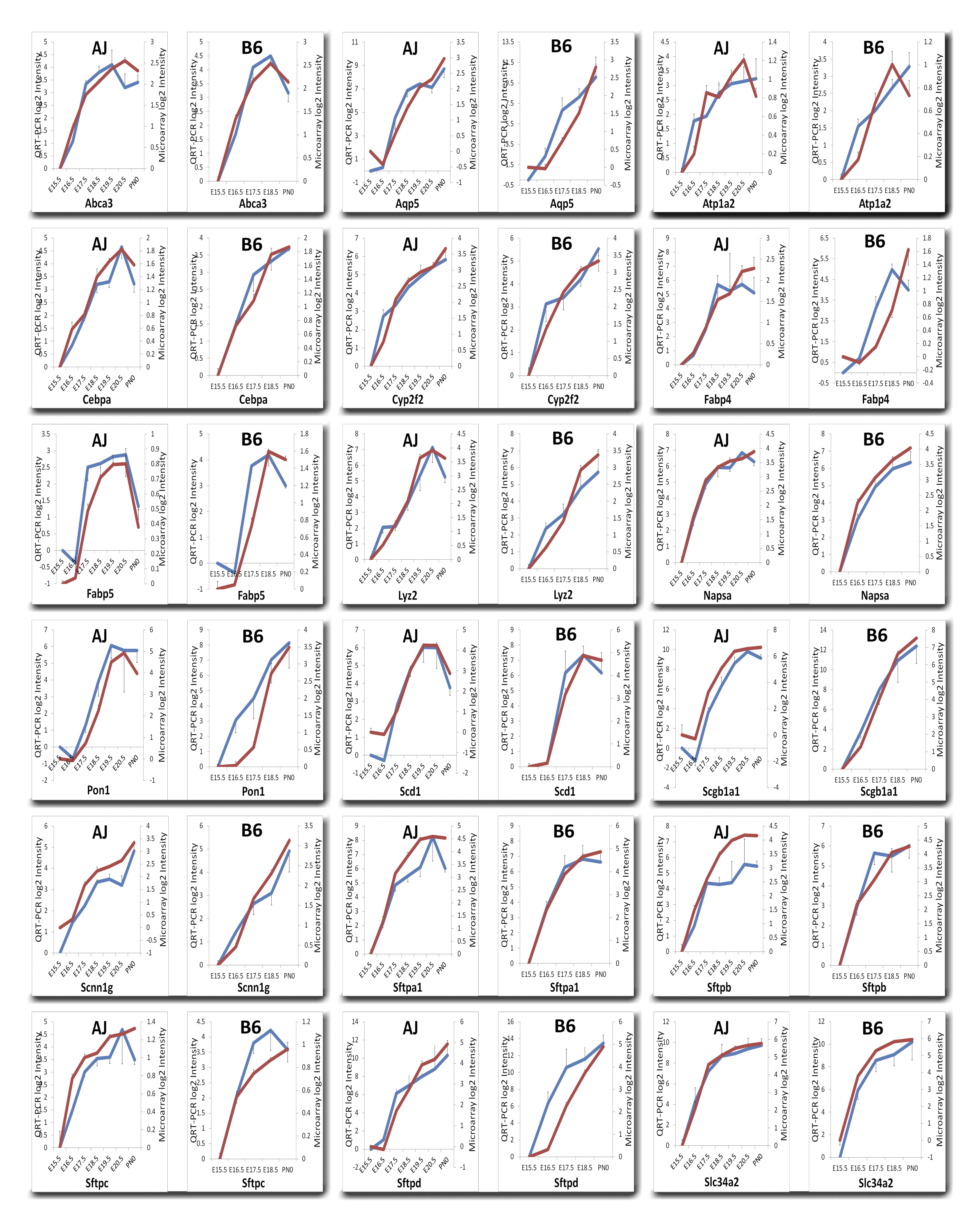

Supplement: Figure S1 — Validation of lung mRNAs of selected genes. The x-axis representing lung samples obtained at each gestational age. The left y-axis is log2 transformed relative mRNA levels determined by QRT-PCR from previous study. The right y-axis is log2 transformed relative mRNA levels determined by mRNA microarray. Red and blue lines represent the expression profiles determined by microarray analysis and QRT-PCR respectively. (TIF) [file pone.0037046.s001.tif]

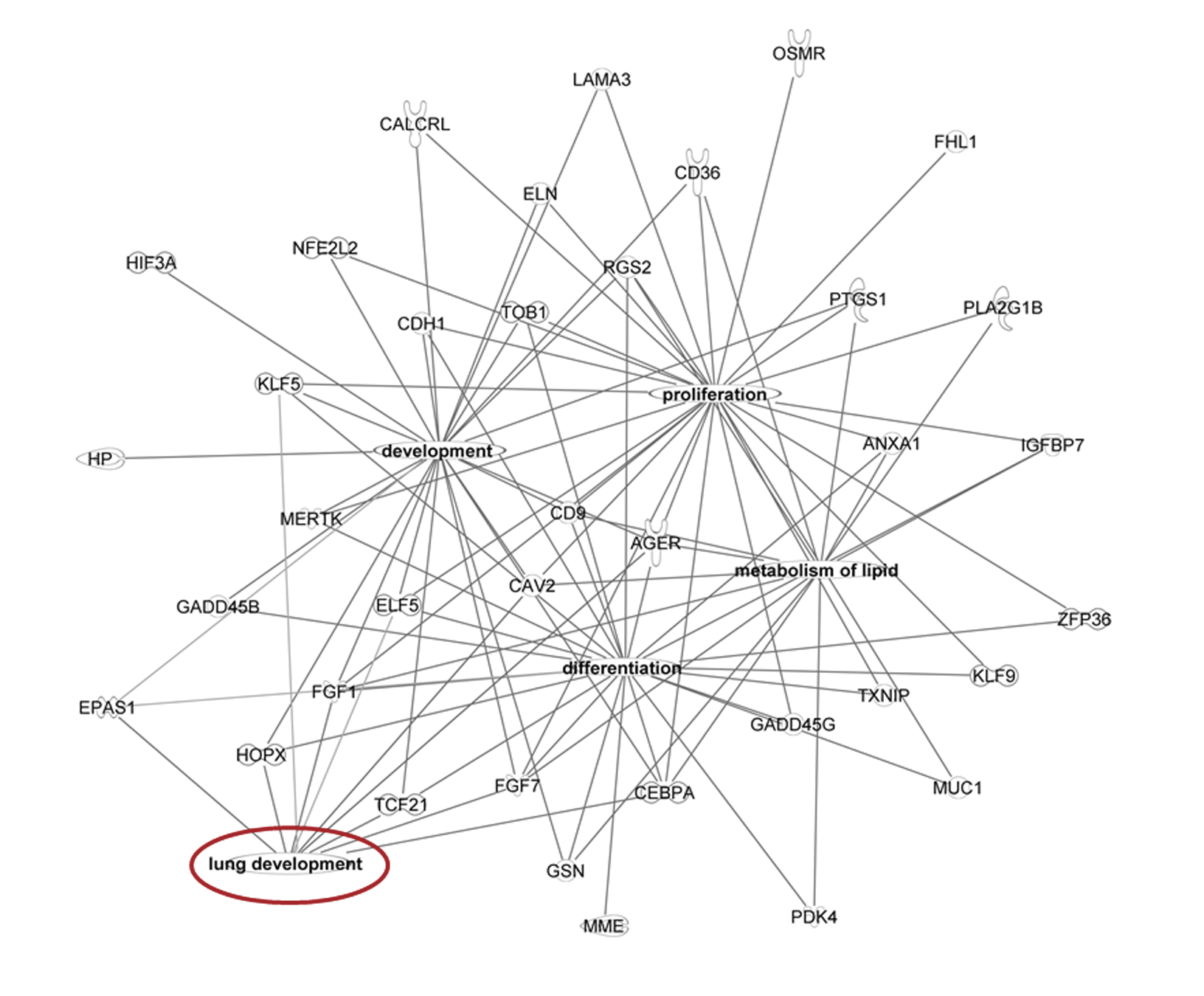

Supplement: Figure S2 — TF/SMs induced from E16.5 were functionally enriched in regulation of cell proliferation and organ development (in particularly lung development). TF/SMs changed during lung maturation were identified by two-way ANOVA and clustered on the basis of their initial change occurring at E16.5, E17.5, and E18.5 or later. The enriched functional categories of TF/SMs induced at different gestation ages were analyzed using Ingenuity pathway Analysis tool (IPA). (TIF) [file pone.0037046.s002.tif]

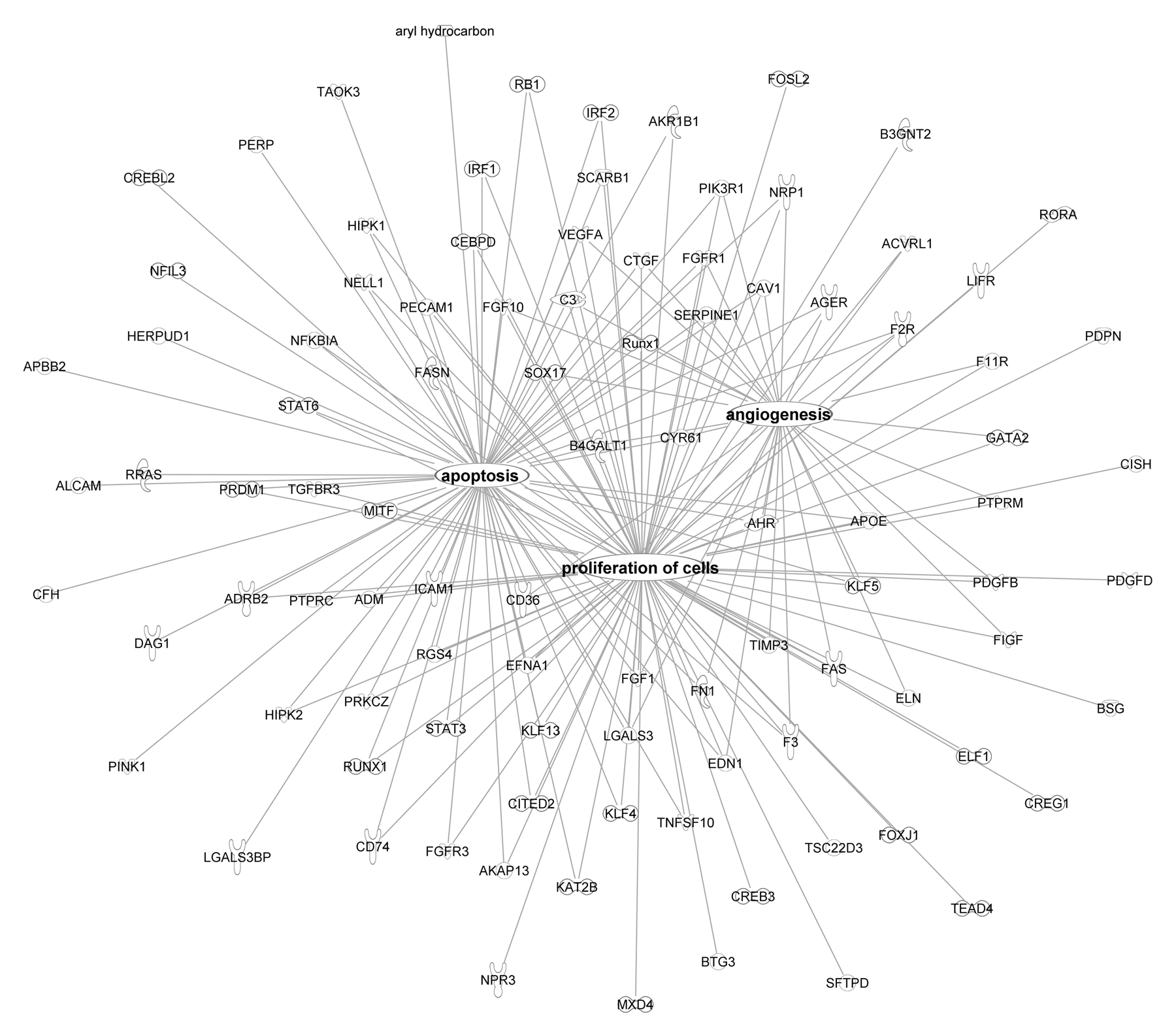

Supplement: Figure S3 — TF/SMs induced from E17.5 were functionally enriched in the regulation of cell proliferation, vasculature development/angiogenesis and apoptosis. TF/SMs changed during lung maturation were identified by two-way ANOVA and clustered on the basis of their initial change occurring at E16.5, E17.5, and E18.5 or later. The enriched functional categories of TF/SMs induced at different gestation ages were analyzed using Ingenuity pathway Analysis tool (IPA). (TIF) [file pone.0037046.s003.tif]

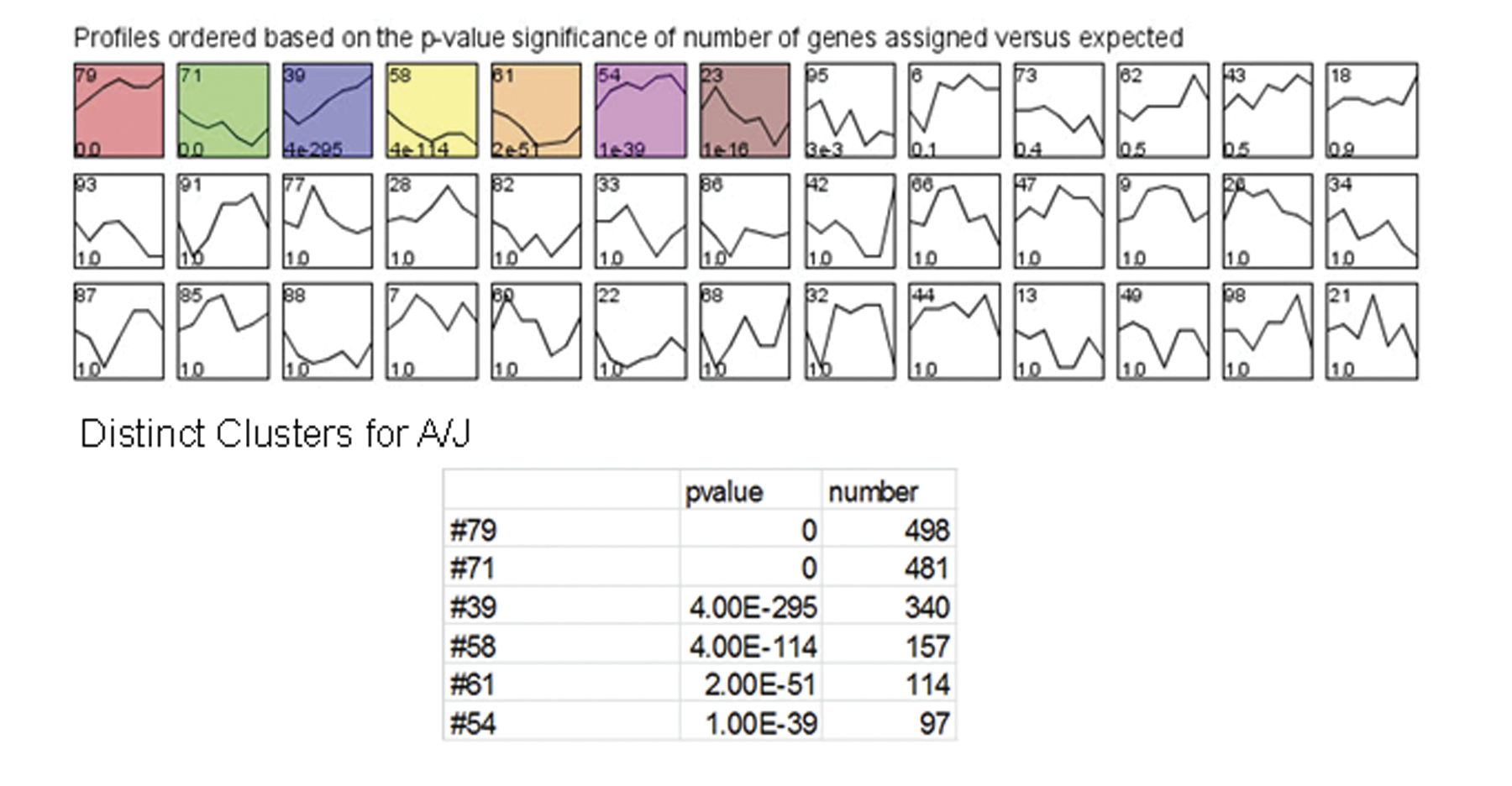

Supplement: Figure S4 — Genes commonly changed during lung maturation were clustered into 7 temporal dependent expression patterns using STEM. (TIF) [file pone.0037046.s004.tif]

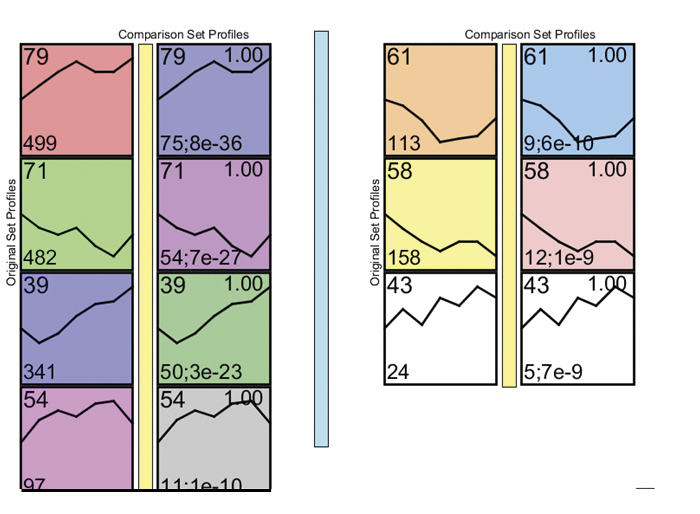

Supplement: Figure S5 — Dynamic expression profiles match of TFSMs and their target genes during lung maturation. We used temporal dependent genes commonly altered in both mouse strains during lung maturation as original set profiles (profiles in the left panel) and TFSMs dynamically changed during lung maturation as comparison set profiles (profiles in the right panel). Significant pattern matches were identified by STEM. (TIF) [file pone.0037046.s005.tif]

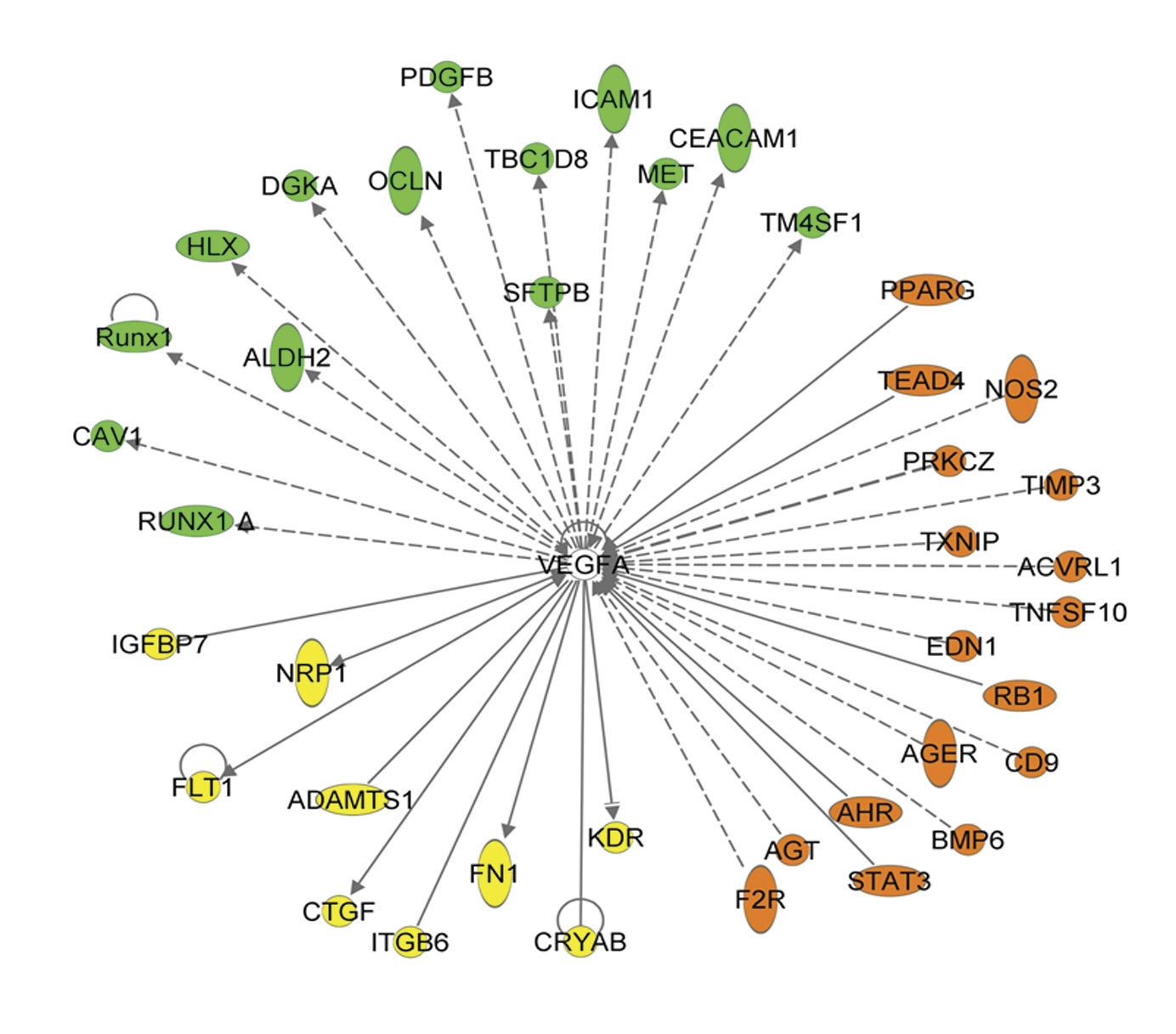

Supplement: Figure S6 — A VEGFA centered sub-network is functionally enriched in mTNAs involved in angiogenesis/vascularization. Yellow nodes are known binding partners of VEGFA. Orange nodes are known to regulate VEGFA expression and green nodes are genes regulated by VEGFA. (TIF) [file pone.0037046.s006.tif]

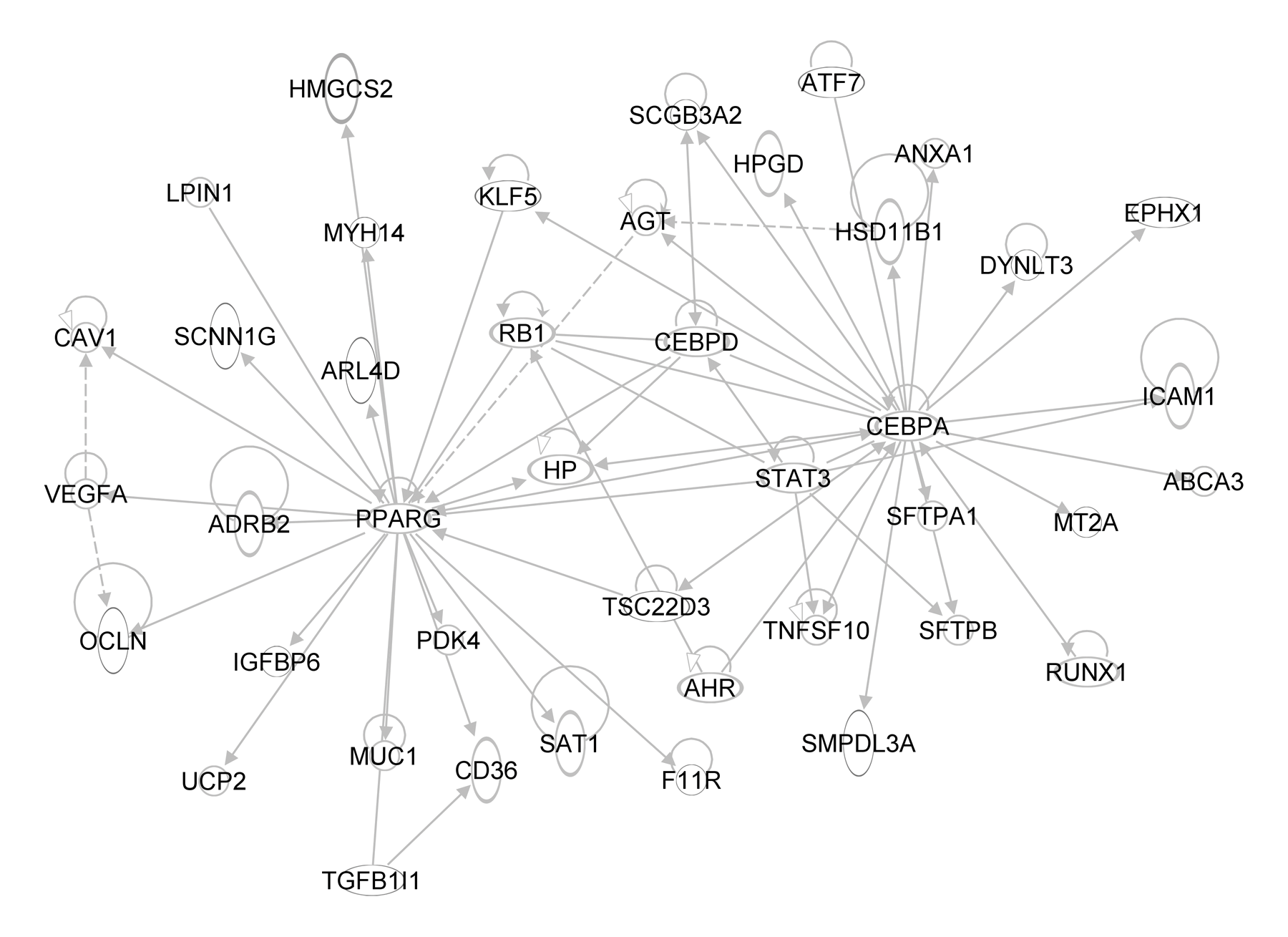

Supplement: Figure S7 — CEBPA-PPARG sub-network regulates lipid metabolism/transport and cell differentiation. Dynamic expression patterns of developmentally changed genes and TF/SMs were matched using STEM. Nearest neighbors of the important hubs from the C79 network were identified and the biological associations of genes in these sub-networks were assessed using Ingenuity pathway Analysis tool (IPA). (TIF) [file pone.0037046.s007.tif]

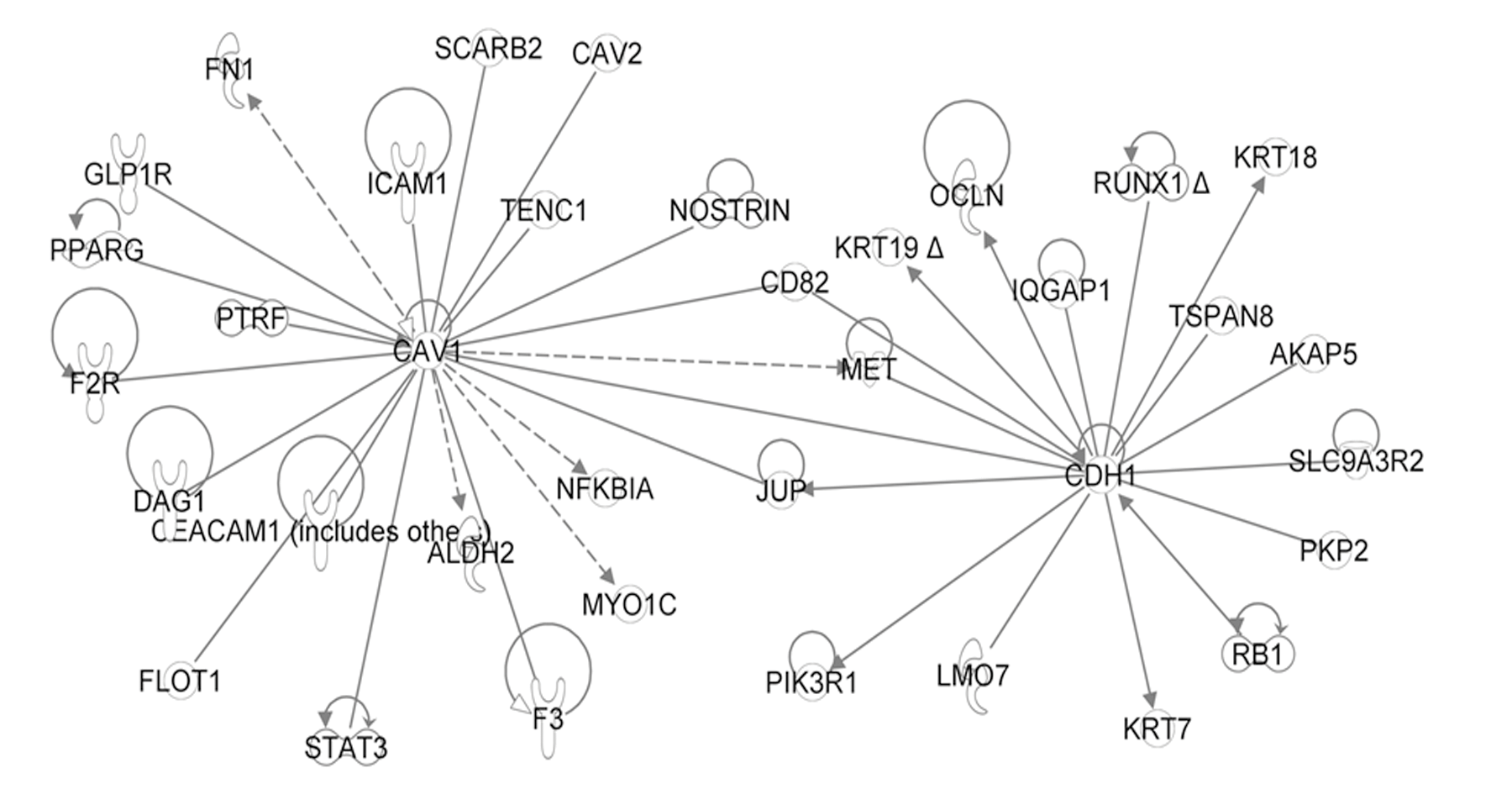

Supplement: Figure S8 — CDH1-CAV1 sub-network regulates cell adhesion, cell movement & tissue development. Dynamic expression patterns of developmentally changed genes and TF/SMs were matched using STEM. Nearest neighbors of the important hubs from the C79 network were identified and the biological associations of genes in these sub-networks were assessed using Ingenuity pathway Analysis tool (IPA). (TIF) [file pone.0037046.s008.tif]

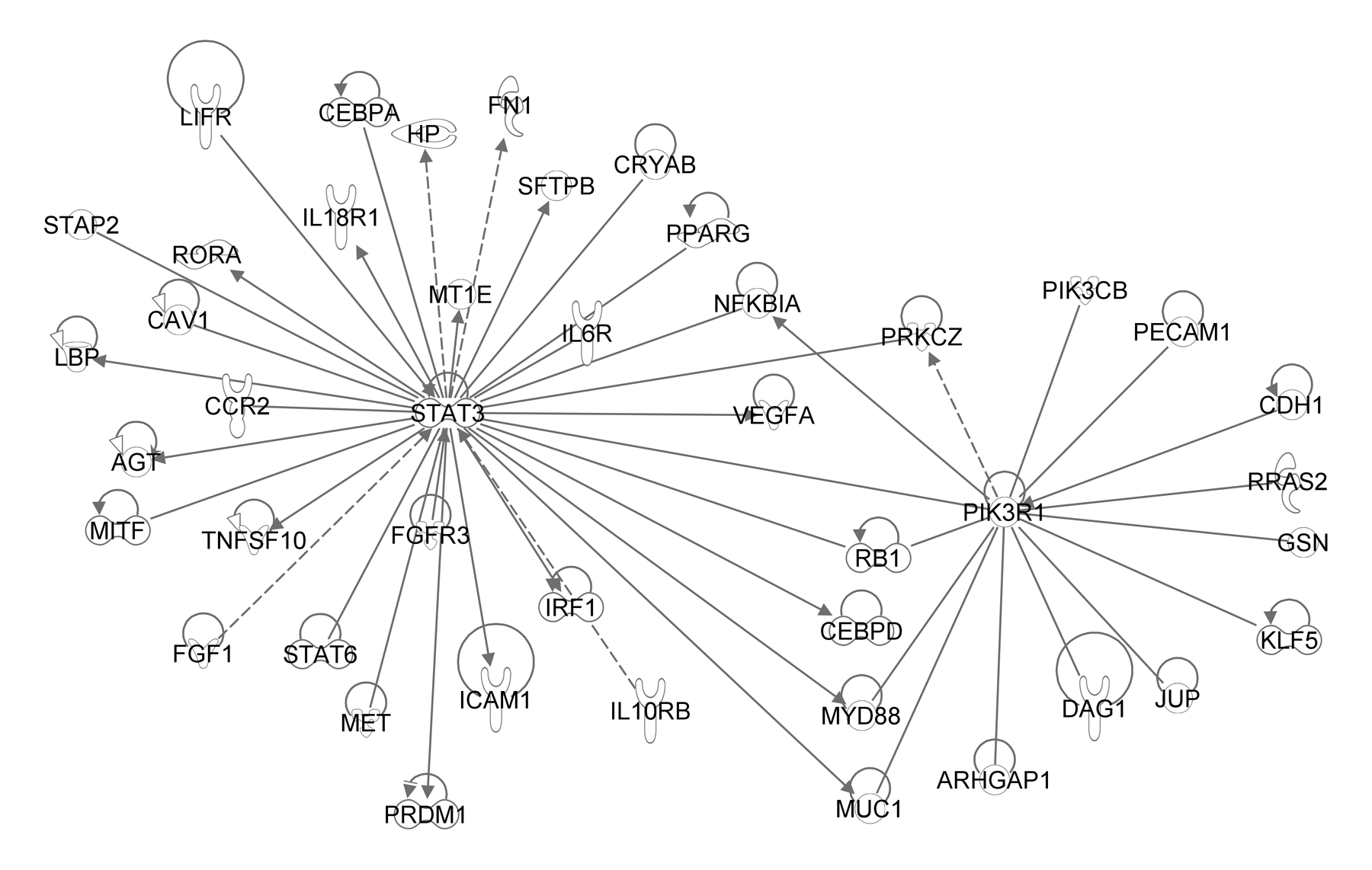

Supplement: Figure S9 — STAT3-PI3K sub-network regulates differentiation, apoptosis and cell proliferation. Dynamic expression patterns of developmentally changed genes and TF/SMs were matched using STEM. Nearest neighbors of the important hubs from the C79 network were identified and the biological associations of genes in these sub-networks were assessed using Ingenuity pathway Analysis tool (IPA). (TIF) [file pone.0037046.s009.tif]

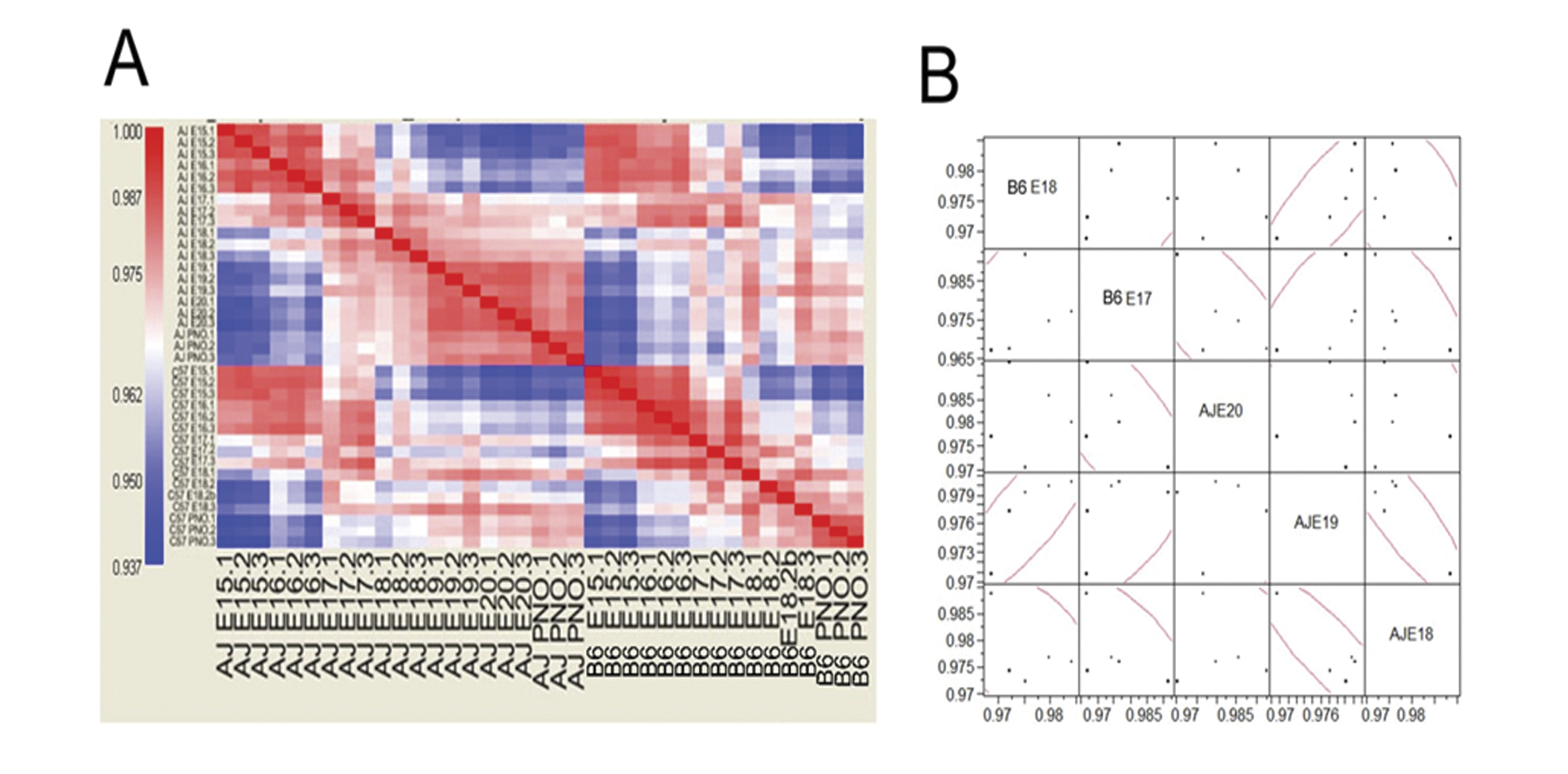

Supplement: Figure S10 — Close correlation of lung mRNAs from B6 (E18.5) and A/J (E19.5). (A) Pearson correlation of lung mRNAs of B6 and A/J mice from E15 to PN0. Each sample was labeled to indicate the strain, age and biological replicates number of the mouse (i.e., AJ E15.1 represents the lung sample from AJ mouse strain at age of E15, litter number 1) (B) Scatterplot of correlation matrix on E18.5 and its neighboring time points. Correlation matrix was generated using multivariate analysis in JMP 9.0 (SAS Institute, Inc. Cary, NC). (TIF) [file pone.0037046.s010.tif]
