# Supplementary material for: Transcriptional Programs Controlling Perinatal Lung Maturation
Source: PLoS One. 2012 Aug 20;7(8):e37046. doi: 10.1371/journal.pone.0037046 (PMC3423373; doi:10.1371/journal.pone.0037046)
Supplement: Table S1 — TFSMs induced from E16.5, peak at E17.5 and decreased thereafter. The dominant expression pattern for TFSMs induced from E16.5 was shown in Figure 2A (i.e., consisting a fast increasing phase from E15–E18 and a slow increasing phase from E18-PN0). There is a small subset of TFSMs induced from E16.5, but peaked at E17.5 and decreased thereafter; genes in this group were known to influence early lung morphogenesis. (DOCX) [file pone.0037046.s011.docx]

Table S1. A subset of TFSMs induced from E16.5, peak at E17.5 and decreased thereafter

| **Gene** | **Evidence** | **PMID** |
| --- | --- | --- |
| Fgfr2 | Required for limb outgrowth and lung-branching morphogenesis. | 16540513 |
| Hhip | Modulates Fgf signaling during lung branching morphogenesis. | 12569124 |
| Hif3a | Abnormal heart development and lung remodeling in mice lacking Hif3a. | 18070924 |
| Lama3 | Proliferation of epithelial cells. | 9950675 |
| Lama5 | Lung development, proliferation of epithelial cells | 12051813 |
| Lpar4 | Protective role in airway injury and remodeling. | 19586906 |
| Nkx2-1 | Regulates lung morphogenesis and differentiation. | 20694477; 12829717 |
| Nr3c1 | Lung maturation, quantity of type I and type II alveolar epithelial cells. | 15265771 |
| Shh | FGF and SHH signaling coordinate lung growth and development | 10518547 |
| Wnt5a | Distal lung morphogenesis. | 12142021 |
