# Supplementary material for: Transcriptional Programs Controlling Perinatal Lung Maturation
Source: PLoS One. 2012 Aug 20;7(8):e37046. doi: 10.1371/journal.pone.0037046 (PMC3423373; doi:10.1371/journal.pone.0037046)
Supplement: Table S2 — Taqman primers used for quantitative RTPCR. Taqman primers (Applied Biosystems Catalog No.) were selected on the basis of microarray experiments examining mouse lung transcript levels during lung maturation. (DOCX) [file pone.0037046.s012.docx]

Table S2. Taqman primers used for quantitative RTPCR.

| Gene | Taqman Primer |
| --- | --- |
| Abca3 | Mm00550501_m1 |
| Aqp5 | Mm00437578_m1 |
| Atp1a2 | Mm00617899_m1 |
| Cbx3 | Mm00850539_g1 |
| Cebpa | Mm01265914_s1 |
| Cyp2f2 | Mm00484087_m1 |
| Fabp4 | Mm00445880_m1 |
| Fabp5 | Mm00783731_s1 |
| Hist1h2ab | Mm00779772_s1 |
| Hist1h2bg | Mm01701376_s1 |
| Hist1h4j | Mm01165508_s1 |
| Hmgb2 | Mm02745766_g1 |
| Lyz2 | Mm01612741_m1 |
| Napsa | Mm00492829_m1 |
| Pon1 | Mm00599936_m1 |
| Scd1 | Mm01197142_m1 |
| Scnn1g | Mm00441228_m1 |
| Sftpa1 | Mm00499170_m1 |
| Sftpb | Mm00455681_m1 |
| Sftpc | Mm00488144_m1 |
| Sftpd | Mm00486060_m1 |
| Slc34a2 | Mm01215846_m1 |
| Hist1h1a | Mm00518898_s1 |

Taqman primers (Applied Biosystems Catalog No.) were selected on the basis of microarray experiments examining mouse lung transcript levels during lung maturation.
